# Adaptability and stability for soybean yield by AMMI and GGE models in Ethiopia

**DOI:** 10.3389/fpls.2022.950992

**Published:** 2022-11-23

**Authors:** Mesfin Hailemariam Habtegebriel

**Affiliations:** ^1^ Department of Microbial, Cellular and Molecular Biology, Addis Ababa University, Addis Ababa, Ethiopia; ^2^ Ethiopian Institute of Agricultural Research (EIAR), Addis Ababa, Ethiopia

**Keywords:** genotype by environment interaction, *Glycine max*., predictability and performance trials, medium set, mega environment

## Abstract

Genotype by environment interaction (GEI) is a phenomenon that occurs in heterogeneous environments that slows breeding progress by preventing the selection of superior cultivars for breeding and commercialization. Therefore, the objectives of this study were to find out how GEI impacts soybean output and to identify the most adapted and stable genotypes. Moreover, to look at the possibility of other mega environments for testing in the future. The experiments were grown for two years in a four-replicated randomized block design at each environment. Over the course of several harvests, yield components, days to flowering, days to maturity, plant height, the number of pods per plants, the number of seeds per plant, hundred seed weight and grain yield per hectare were evaluated in the main for 2018 and 2019.To analyze the stability performance of the genotypes, general linear method, GGE and Additive main effect and multiplicative interaction effects analysis (AMMI) and ASV rank analysis were applied. The GGE biplot revealed that the GGE biplots explained 74.29% of the total variation distributed as,56.69% and 17.62% of sum of squares between principal component PC1 and PC2, respectively whereas, AMMI model, the first two interaction principal component axes (IPCA1 and IPCA2) explained 47.74% and 26.62% of the variation due to GEI, respectively, exposed genotypes identified the five as best performer. The results from the four distinct stability statistics AMMI biplot (G8, G2, G1, G11), ASV (G1, G11; (GSI; G9, G1, G11) and (GGE: G2, G8, G9) are taken into account together with the genotypes` grand mean. The genotypes JM-CLK/CRFD-15-SD (G8) and 5002T (G1), which rank among the best and have the highest seed output, are suitable for hybridization as a parent and commercial production. Therefore, genotypes JM-CLK/CRFD-15-SD (G8) and 5002T(G1) have the highest seed output were among the best and thus could be recommended for release as a new soybean varieties cultivation across.

## 1 Introduction

Cultivated soybean [*Glycine max (L.) Merrill*] is a self-pollinating and an important leguminous source of food, feed, fuel and oil crop with high commercial value widely cultivated globally (Bocianowski et al., 2019; Sritongtae et al., 2021 and Abebe et al., 2021). First, the annual wild soybean (*Glycine soja*), the kindred ancestor of the current cultivated soybean (*Glycine max*), originated and is found throughout China (Qiu and Chang, 2010). In Ethiopia, it is commonly known “*akuri ater”* in *Amharic, “akuri bonsitu”* in Oromic, imported for tackling for multi purposes problems in early 1950’s. The crop also thrives in places as low as 500 m.a.s.l and as high as 1900 m.a.s.l. that get a well-distributed average rainfall of 550 to 700 mm over the growing year.

In addition, a lack of soybean genotypes suitable for growth in diverse conditions as soybean breeders have developed their own varieties for specific purposes (Sritongtae et al., 2021). Similarly, Oladosu et al. (2017) and Mushoriwa et al. (2022) also reported that the GEI reveals the challenges that plant breeders have, when identifying a superior genotype for commercial farming before releasing it as a variety. Due to the fact that analysis of variance (ANOVA) cannot adequately shed light on the genotypes or environments that contribute to the interaction (Oladosu et al., 2017; Khan et al., 2021). In general, the knowledge and extent of the GEI provided by this study would aid breeders in allocating limited resources to the appropriate varietal development cycle, as a phenotype is a combination of genotype (G) and environment (E) components, as well as a GEI. Plant breeders conduct multi-environment experiments (MET) to examine the yield stability performance of genetic resources under diverse environmental circumstances (Yan and Hunt, 2002; Mwiinga et al., 2020).

Many researchers and breeders have investigated and reported grain yield stability using a variety of soybean genotypes in various agro-climatic zones (Samyuktha et al., 2020; Mwiinga et al., 2020; Happ et al., 2021), are useful for evaluating crop yield stability across varied environmental circumstances and determining acceptable habitats for all genotypes studied. Many stability methods, such as regression, have been devised to uncover the GEI pattern (Eberhart and Russell, 1966), but AMMI and GGE were by far providing the better interpretation of GEI data and widely used than the rest of stability procedures (Bhartiya et al., 2017; Khan et al., 2021).The AMMI technique combines ANOVA and principal component analysis with a biplot displaying the genotype means and their relationship to the first PCA as a key output (Mitrović et al., 2012; Bocianowski et al., 2019). The GGE biplot will aid researchers in better understanding complicated GEIs in multi-environment breeding line trials and agronomic investigations (Luo et al., 2015).

Phenotypic selection, one of the most critical processes in genetic improvement, is the foundation of traditional soybean breeding. According to Mederos-Ramirez et al. (2021), GEI in the improvement programs has a determining influence on the new cultivar obtaining and more stable and adapted genotypes. However, several studies on the adaptation and stability of soybean genotypes (Gurmu et al., 2009; Krisnawati et al., 2018a; Happ et al., 2021; Mushoriwa et al., 2022). So far, several statistical models have been proposed to analyze feature stability and GEIs through traditional analysis. The AMMI and the genotype and genotype x environmental interaction effect (GGE) model can detect GEIs in terms of crossover effects due to significant changes in ranking. Therefore, it is widely used. The result is the surrounding genotype. In addition, AMMI and GGE have proven to be particularly useful in visualizing GEI effects in graphic representations. Therefore, the objectives of this study were (i). to investigate how GEI affects in the soybean yield using AMMI and GGE analysis and (ii). identification of most adapted and stable, best-performing genotypes that can recommend as a variety to potential customers. Furthermore, to investigate the existence of other mega-environments in order to determine the optimum test environments for future testing.

## 2 Materials and methods

### 2.1 Plant genetic materials, trial planning and execution

Nine elite soybean lines developed by Jimma Agricultural Research Centre, the national soybean program, and SCS-1 and Nyala were used as control (**Table 1**) were used in the study. Over two years, eleven environments, experiments with genotypes were performed in a randomized complete block design (RCBD) with four replicates was used to conduct the genotype experiment over each year. The plot size was 4 x 1.6 m, there were four rows and the spacing between rows was 60 cm and between plants was maintained at 5 cm. Two seeds per hill were planted, and after 2-3 weeks, the hill was reduced to one plant per hill. The NPS and Urea fertilizer was applied at the recommended amount of 122 kg ha^-1^ and 50 kg ha^-1^ during sowing and flowering and respectively. Weeds were controlled three times, remainder of the agronomic management practices like pest or diseases control was done as required.

**Table 1:** Description of the eleven soybean genotypes used in this study.

Genotype	Pedigree	Source	Maturity	Remark	Growth Habit	Generation
G1	5002T	USA introduction	Medium	Promising line	Indeterminate	F8
G2	SCS-1	CIMMYT-Zimbabwe	Medium	Released	Indeterminate	Control
G3	Ozark	USA – introduction	Medium	Promising line	Indeterminate	F8
G4	KS4895	USA –introduction	Medium	Promising line	Indeterminate	F8
G5	Harber	USA – introduction	Medium	Promising line	Indeterminate	F8
G6	JM-PR142/CLK-15-SE	Ethiopia- Cross	Medium	Promising line	Indeterminate	F8
G7	Hs93-4118	USA –introduction	Medium	Promising line	Indeterminate	F8
G8	JM-CLK/CRFD-15-SD	Ethiopia- Cross	Medium	Promising line	Indeterminate	F8
G9	PI471904	USA- introduction	Medium	Promising line	Indeterminate	F8
G10	PI417089A	USA- introduction	Medium	Promising line	Indeterminate	F8
G11	Nyala	Ethiopia –released	Medium	Released	Indeterminate	Control

### 2.2 Study area

**Figure 1:**
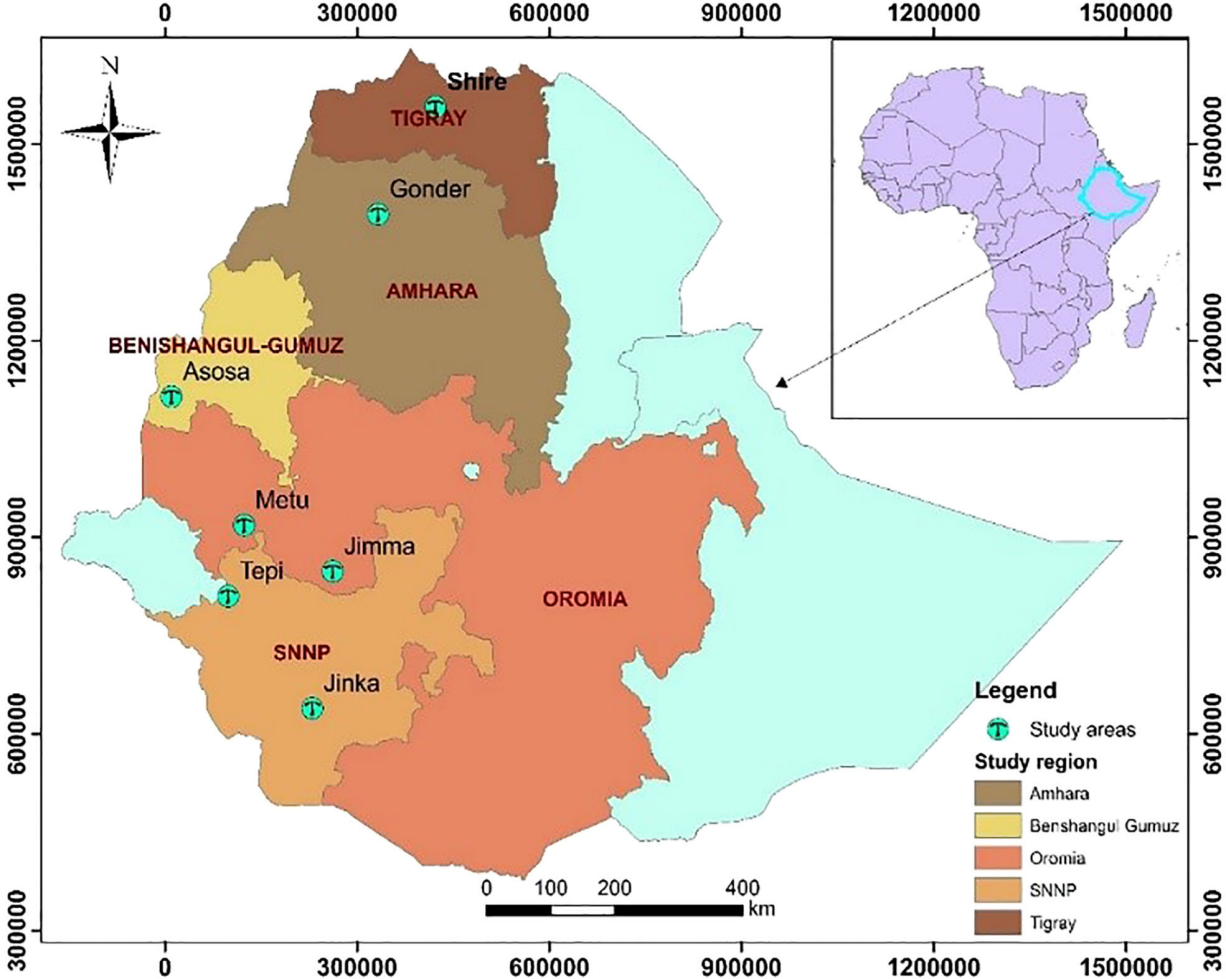
Geographical map (constructed using geographic information system (ArcGIS) showing testing environments: Jimma and Metu are situated Oromia regional state, Shire is located in Tigray regional state, Gondar situated Amhara regional state, Jinka and Tepi are located in southern nation, nationalities and peoples Regional State, Asosa (2018-2019) is situated Benishangul Gumez regional states.

The trials were conducted over two years in 2018 and 2019 at Jimma (L1), Metu (L2), Asosa (L3), Shire (L4), Gondar (L5), Jinka (L6), and Tepi (L7). The trials were planted throughout two years in 2018 and 2019, but at Gondar, Jinka, and Tepi, the trials were only planted in 2019 (See **Figure 1**) with the total descriptions below (**Table 2**).

**Table 2:** The eleven sites and soil attributes used for evaluation across the country are described.

Year Designations	Environment	Year	Latitude(N)	Longitude(E)	Elevation (m.a.s.l)	Rainfall (mm)	Sunshinehour (hr.)	Averagetemperature (o c)	R.H(%)	Soil type
Y1L1	Jimma	2018	36^0^82’	7^0^67’	1775	1561	6.8	9-28	65.1	Chromic Nitosol and Combisol
Y1L2	Metu	2018	35^0^57’	8^0^28’	1550	1835	–	12.5-28.6	82	Reddish brown
Y1L3	Asosa	2018	34^0^52’	10^0^00’	1580	1,236	7	14- 39	58	Dystric Nitosols
Y1L4	Shire	2018	38^0^29’	14^0^10’	1871	905	7.8	17.57	52.6	Cambisols
Y2L1	Jimma	2019	36^0^82’	7^0^67’	1775	1561	5.6	9-28	74.9	Chromic Nitosol and Combisol
Y2L2	Metu	2019	35^0^57’	8^0^28’	1550	1835	–	12.5-28.6	82	Reddish brown
Y2L3	Asosa	2019	34^0^52’	10^0^00’	1580	1,236	8	16.5- 27.5	38	Dystric Nitosols
Y2L4	Shire	2019	38^0^29’	14^0^10’	1871	905	7.8	12.5-28.2	46	Cambisols
Y2L5	Gondar	2019	37^0^43’	12^0^52’	1973	912	7.7	19.8-26.1	56.1	Vertisol
Y2L6	Jinka	2019	36^0^55’	5^0^77’	1375	162.9	5.5	15.56-29.6	67.3	Nitosol
Y2L7	Tepi	2019	35^0^44’	7^0^20’	1208	1559	–	15.5 -29.7	78	Nitosol

m.a.s. l, meter above sea level; mm, millimetres; hr, hours; o ^c^, degree centigrade; %, percent; R.H, Relative humidity.

### 2.3 Evaluation of morphological traits and method of phenotypic data collection

All phenotypic data were collected from 10 randomly selected and tagged plants in each plot in each replication at various growth stages in the field and the field crops laboratory after harvest according to the soybean descriptor (1986). Days to flowering, days to maturity, plant height (cm), the number of pods per plant, the number of seeds per plant, and hundred seed weight (gm) were all reported as agronomic parameters.

### 2.4 Statistical analysis

#### 2.4.1 Analysis of variance

Prior to proceeding with the analysis of variance (ANOVA), a homogeneity test was conducted, and then all the data considered were subjected to a combined analysis of variance over their environment, which was performed using the SAS program software with the following the combined ANOVA model equation:


(Combined ANOVA model) ${\text{Y}}_{\text{ijkl}}=\mu +G_{i}+E_{j}+Rk_{\left(j\right)}+Bl(jk)+GE_{ij}+\epsilon_{ijkl}$

Where *Y_ijkl_* is the response of the ith genotype in jth environment and *k^th^* replication within environment and *l^th^* block within replication; μ is the grand mean, *G_i_* is the genotype effect i; *E_j_* is the environment effect j; *Rk_j_* is the replication within environment effect *k*;*Bl_jk_* is the block within replication effect l; *GE_ij_* is the genotype × environment interaction effect; and *ε_ijkl_* is the random error.

#### 2.4.2 Additive main effects and multiplicative interaction (AMMI) analysis and AMMI’s stability value (ASV)

AMMI analysis was carried out using the software GEA-R version 4.1 (Angela et al., 2015) with the model equation:


(AMMI model) $${\text{Y}}_{\text{ger}}{\text{=μ+α}}_{\text{g}}{\text{+β}}_{\text{e}}+\sum_{n=1}^{\text{N}}{\text{λς}}^{\text{gn}}{\text{η}}_{\text{en}}{\text{+ρ}}_{\text{ge}}{\text{+ϵ}}_{\text{ger}}$$

where:

**Y_ger_=**the grain yield level for genotypes g in environment e for replicate r

μ = the grand mean

***α_ɡ_* =** genotype mean deviations (mean minus the grand mean)

***β_e_* =**the environmentsmean mean deviations

**N=**the number of singular value decompostion(SVD) axes retained in the model

***λ_n_* =** the singular value for SVD axis

**η*ς_ɡn_* =** the genotype of singular vector values for SVD axis ***n***

***θ_ɡe_* =**the interaction residuals

***ρ_ɡe_* =**The AMMI residuals

***ε_ɡer_* =**Error term mean

$\sum_{n=1}^{\text{N}}{\text{λς}}^{\text{gn}}{\text{η}}_{\text{en}}{\text{+ρ}}_{\text{ge}}{\text{+ϵ}}_{\text{ger}}$ is equivalent to the interaction term in the ANOVA model.

The AMMI stability value (ASV) as described by Purchase et al. (2000), was calculated as follows: $\text{ASV}=\sqrt{{\left[\frac{\left(SS_{IPCA1}\right)}{\left(SS_{IPCA2}\right)}(IPCA_{1})\right]}^{2}+{\left(IPCA_{2}\right)}^{2}}$ , where, SS is the sum of squares, IPCA1 and IPCA2 are the first and second interaction principal component axes, respectively; and the IPCA1 and IPCA2 scores were the genotypic scores in the AMMI model. ASV is the distance from zero in a two-dimensional scatterplot of IPCA1 scores against IPCA2 scores.

Another parameter that we can calculate in AMMI analysis is the genotype selection index (GSI). Selection for stability will not necessarily lead to the best genotype. Thus, the GSI for each genotype was computed *via* the sum of the rank of the genotype grain yield (RYi) and the rank of the genotype ASV (RASVi). The most stable genotype was suggested to be the one with the lowest GSI (Farshadfar et al., 2012).

#### 2.4.3 GGE analysis

If there is a significant difference in genotype–environment interaction, the GGE biplot method will be employed to analyse and assess the interaction and yield stability (Olanrewaju et al., 2021). A general model of GGE biplot based on environment-centric or environment-standardized singular value decomposition (SVD) can be written as:


$${\text{Y}}_{\text{ij}}{\text{=μ+β}}_{\text{j}}{\text{+λ}}_{1}{\text{ξ}}_{i1}{\text{η}}_{j1}{\text{+λ}}_{2}{\text{ξ}}_{i2}{\text{η}}_{j2}{\text{+ϵ}}_{\text{ij}}$$

Where, *Y_ij_*, is the trait mean for genotype i in environment j; µ is the grand mean; *β_j_* is the main effect of environment j; µ + *β_j_* is the mean yield across all gen’genotypes in environment j; λ1 and λ2 are the singular values (SV) for the first and second principal components), respectively; *ξ_i1_* and *ξ_i2_* are eigenvectors of genotype *i*for PC1 and PC2, respectively; η1j and η2j are eigenvectors of environment j for PC1 and PC2; and *ξ_ij_* is the residual associated with genotype *i* in environment *j*. In GGE biplot analysis, scores of PC1 were plotted against PC2.

## 3 Results

### 3.1 Combined analysis of variance for yield and related traits

For the majority of the factors, the results of the analysis of variance show that there is a substantial difference between genotypes, environment and genotypes x environment (P<0.001) (**Table 3**). The yield production of the examined soybean genotypes ranged from 1.70 t ha^-1^ to 2.84 t ha^-1^, according to the combined data analysis results. The average grain yield of the control variety was 2.62 t ha^-1^. The genotypes JM-CLK/CRFD-15-SD had a maximum grain yield of 2.85 t ha^-1^, while the genotypes Hs93-4118 had the lowest grain yield of 1.70 t ha^-1^, respectively. The genotypes 5002T and SCS-1 yielded more than the control variety.

**Table 3:** Estimation of significant level for yield and yield contributed traits for eleven soybean genotypes revealed by ANOVA.

SOV	df	DTF(Days)		DTM (Days)		PH (cm)		NPP		NSP		HSW (gm)		Yield (t ha^-1^)	
		MS	TSS (%)	MS	TSS (%)	MS	TSS (%)	MS	TSS (%)	MS	TSS (%)	MS	TSS (%)	MS	TSS (%)
**Replications (R)**	3	58.5ns	0.71	18.1ns	0.13	57.9 ns	0.34	23.3 ns	0.20	362.5ns	0.080	1.9 ns	0.38	0.5 ns	0.79
**Locations (L)**	6	2804.4**	34.06	11980.6**	86.92	5162.5**	30.75	69.7**	0.61	126832.9**	27.889	238**	47.96	11.7**	18.46
**Genotypes (G)**	10	1241.7**	15.08	584.0**	4.24	7151**	42.59	768.7**	6.76	4198.1**	0.923	120.5**	24.28	3.0**	4.73
**GEI**	30	175.3**	2.13	95.7**	0.69	180.3**	1.07	182.1**	1.60	1914.7**	0.421	7.2ns	1.45	1.3**	2.05
**Error**	261	54.6**	0.66	20.7**	0.15	34.9**	0.21	61.3**	0.54	451.6**	0.099	2.2**	0.44	0.20**	0.32

SOV, Source of variations; df, degree of freedom; TSS (%), Total sum of squares; DTF, Days to flowering; DTM, Days to maturity; NPP, Number of pods per plant; NSP, Number of seeds per plant; HSW, Hundred seed weight; **, Significant at 0.01.

To explain the main effect and quantify the interactions among and within the sources of variability, a combined analysis of variance was performed. The findings of the pooled analysis of variance are shown in **Table 3**. Significant variations (p≤ 0.01, p ≤0.05) were found in the mean square of environments, genotypes, and GEI for DTF, DTM, and NSP. For genotypes by environment interaction (GEI), all but one variable had significant variance, while the trait had non-significant variation for genotypes by environment interactions (GEI). Large differences in environments, and genotypes could be attributed to differences in environmental conditions and genetic makeup that differ from one place to the next. **Table 3** shows the percentages of variation for all attributes when the proportion of GEI (% of GEI) is partitioned using the whole sum of the squares. Other attributes exhibited a large range of variance related to environment, ranging from 0.61 to 86.92%, with the exception of the total number of pods (NPP). The majority of genotype performance variance is attributable to a greater disparity in genotype means between sites. Genotype by environment variation was found to be lower, ranging from 0.42 to 2.13%. Days to flowering (15.08%), plant height (42.59%), and hundred seed weight (24.28%) all contributed nearly the same amount to genotype effect, whereas days to maturity (4.24%), the number of pods per plant (6.76%), number of seeds per pod (0.923%), and yield per hectare (4.73%) all contributed very little. The genotype by environment interaction (GEI) is significant for almost all variables, including days to flowering, days to maturity, plant height, number of pods per plant, number of seeds per plant, and grain yield per hectare, but hundred seed weight.

#### 3.1.1 Mean performance and comparison of genotypes

The means comparison and average performance of eleven soybean genotypes over eleven environments were listed in **Table 4**. Over the environment, all the genotypes showed significant variation for days to flowering, days to maturity, plant height, the number of pods per plant, the number of seeds per plant, hundred seed weight (HSW), and yield per hectare. The days to flowering ranged from 51.4 (5002T) to 63.3 (PI471904 and SCS-1) with an average of 55.9 days. The genotype PI471904 produced the highest number of days to maturity (122.5) followed by SCS-1 (119.71), JM-CLK/CRFD-15-SD (117.9), and JM-PR142/CLK-15-SE (117.7) though across the genotype it was 106 while the lowest was 51. The highest plant height was 86.8 cm for genotype PI471904, followed by PI417089A (81.5 cm), and the lowest was 44.4 cm for the genotype Ozark. The greater value of hundred seed weight was accounted for 21.6 g (5002T) followed by 21.0 g (PI417089A) though the lower value was 14.1 g for PI471904. The average weight of hundred seeds was 18.4 g with a range of 14.1 g to 21.0 g over tested environments. In terms of yield per hectare, genotype JM-CLK/CRFD-15-SD had the highest yield (2.84 t ha^-1^), followed by genotypes 5002T (2.73 t ha^-1^) and PI471904 (2.64 t ha^-1^), while genotype Hs93-4118 had the lowest yield of 1.7 t ha^-1^. However, yield per hectare varied from 1.70 to 2.84 t ha^-1^, with an average of 2.4 t ha^-1^ throughout the studied environments.

**Table 4:** Mean performance days to flowering, days to maturity, plant height, number of pods per plant and, number of seeds per, branch per plant early maturing across years and environments in 2018 and 2019.

N°	Genotype	Yield (t ha^-1^)													Combined value of yield related traits					
		Y1L1	Y1L2	Y1L3	Y1L4	Y2L7	Y2L1	Y2L2	Y2L3	Y2L4	Y2L5	Y2L6	Overall	DF		DM	PH (cm)	NPP	NSP	HSW (gram)
1.	5002T	2.97	2.61	1.64	3.72	2.24	2.82	2.90	3.70	2.89	2.18	2.40	2.73	51.4		114.6	64.1	29.6	70.5	20.6
2.	SCS-1	2.95	1.97	1.31	4.72	2.26	1.92	2.09	4.00	3.03	1.92	2.12	2.57	63.3		119.7	64.7	38.8	87.0	17.1
3.	Ozark	2.58	2.24	0.97	3.07	1.76	2.87	2.97	2.26	2.75	2.02	2.54	2.37	50.7		112.3	44.4	28.3	59.1	18.3
4.	KS4895	2.22	2.08	1.28	3.02	1.97	2.37	2.21	2.45	2.80	1.52	2.80	2.25	51.7		110.5	46.7	29.2	65.0	16.9
5.	Harber	2.31	1.70	1.17	2.65	1.95	2.58	2.30	2.42	2.39	1.32	2.21	2.09	51.2		111.6	45.3	30.9	69.3	18.8
6.	JM-PR142/CLK-15-SE	2.33	2.39	1.36	3.29	2.03	1.36	3.05	2.66	2.71	1.91	2.25	2.30	60.9		117.7	62.3	37.2	76.9	18.8
7.	Hs93-4118	1.40	1.19	0.40	2.30	1.59	2.39	1.84	1.31	2.97	1.28	2.04	1.70	45.2		108.1	42.4	27.6	62.3	18.6
8.	JM-CLK/CRFD-15-SD	3.05	2.72	1.91	3.77	2.47	2.37	2.98	4.27	2.91	2.17	2.62	2.84	62.8		117.9	64.5	36.7	78.8	18.4
9.	PI471904	1.76	2.60	1.53	3.74	3.24	1.53	2.41	3.06	3.18	2.96	3.03	2.64	63.3		122.5	86.8	42.2	96.2	14.1
10.	PI417089A	2.47	2.26	1.50	2.62	1.82	2.66	2.61	3.22	2.60	1.79	1.80	2.30	56.5		114.0	81.5	35.1	78.8	21.0
11.	Nyala(C1)	2.56	2.44	1.70	2.58	2.44	1.71	2.32	3.71	1.83	1.80	2.71	2.35	57.5		115.9	53.4	30.9	62.8	20.2
Mean		2.4	2.2	1.3	3.2	2.2	2.2	2.5	3.0	2.7	1.9	2.4	2.4	55.9		115.0	59.6	33.3	73.3	18.4
Min. across environments		1.4	1.2	0.4	2.3	1.6	1.4	1.8	1.3	1.8	1.3	1.8	1.7	45.2		108.1	42.4	27.6	59.1	14.1
Max. across environments		3.1	2.7	1.9	4.7	3.2	2.9	3.1	4.3	3.2	3.0	3.0	2.8	63.3		122.5	86.8	42.2	96.2	21.0
CV (%)		12.83	16.92	19.66	20.28	19.83	12.46	17.47	19.19	14.37	30.27	34.18	18.8	13.3		4.0	9.9	23.5	29.0	8.1
LSD _0.05_		0.41	0.61	0.38	0.79	0.61	0.43	0.63	0.83	0.66	0.82	1.18	0.21	3.6		2.2	2.9	3.9	10.5	0.7

Jimma (L1), Metu (L2), Asosa (L3), Shire(L4), Gonder(L5), Jinka(L6), and Tepi(L7), DF, Days to flowering; DM, Days to maturity; Plant Height(cm), NPP, Number pf pods per plant; NSP, Number of seeds per plant; HSW, Hundred seed weight(gm).

### 3.2 AMMI model analysis for grain yield

According to the results of the AMMI model’s analysis of variance, genotype, environment, and GEI all had a significant (p<0.001) impact on grain yield, explaining 16.18%, 49.42%, and 33.91% of the variation, respectively. It also revealed three PCs with highly significant differences (p<0.001) and the first three interaction principal component of AMMI, explaining 47%, 26.62%, and 17.88% of the GEI with 19, 17, and 15 degrees of freedom (df), respectively, as well as the fourth PC with significant differences (p<0.05) explaining 7.77% of the interaction with the degree with total cumulative of nearly 100%. The environment explains a major amount of the yield differential, showing that the environments were different, according to AMMI.

### 3.3 IPCA scores, AMMI stability values and mean yields

According to this parameter, lines G6, G1, G10, G5 and G11 had lowest stability values of ASV, identified as stable lines. On contrary, genotypes G8, G7, G2, G4 and G3 had the highest stability value hence are less stable. GSI integrates both yield and stability across environments. Genotypes with lower GSI (G9, G1 and G11) were desirable since they combine high mean yield performance with stability. Three out of three had higher stability than the average site of 2.4 t ha^-1^; G9 had a mean performance ranking of 2.64 t ha^-1^, followed by G1 and G11, which have 2.53 t ha^-1^ and 2.48 t ha^-1^, respectively. Since these three genotypes had above the average yield, we can consider as the stable and adapted genotypes for the wider productions. And, similarly for the environments Asosa19, Shire18, Shire19, Jimma18 and Jinka19 were stable places, which is less discriminatory. In addition, Shire19 which had the highest yield of 3.24 t ha^-1^ (**Table 5**).

**Table 5:** Average grain yield, IPCA score, ASV and GSI Soybean genotypes across sites.

Genotype*s* Name	Code	Mean	RY_i_	IPCA1	IPCA2	ASV	ASV Rank(RASVi)	GSI
5002T	G1	2.75	4	0.37	-0.36	0.53	2	6
PI417089A	G10	2.30	7	-0.09	-0.53	0.53	3	10
Nyala (C1)	G11	2.35	5	0.56	-0.29	1.11	5	10
SCS-1	G2	2.56	3	0.74	0.11	5.12	9	12
Ozark	G3	2.37	6	-0.61	-0.26	1.43	7	13
KS4895	G4	2.24	9	-0.39	0.03	4.82	8	17
Harber	G5	2.09	10	-0.39	-0.33	0.56	4	14
JM-PR142/CLK-15-SE	G6	2.30	7	0.09	0.26	0.26	1	8
Hs93-4118	G7	1.70	11	-0.98	0.19	5.12	10	21
JM-CLK/CRFD-15-SD	G8	2.75	1	0.64	-0.02	19.96	11	12
PI471904	G9	2.64	2	0.06	1.22	1.22	6	8
**Environments Name**								
Y1L1	Jimma18	2.14	8	2.14	-0.96	4.84	1	9
Y1L2	Mettu18	2.52	4	2.52	-0.22	29.25	8	12
Y1L3	Asosa18	1.30	11	1.30	0.11	15.53	5	16
Y1L4	Shire18	2.72	3	2.72	-0.52	14.32	3	6
Y2 L1	Jimma19	2.45	5	2.45	0.31	19.47	6	11
Y2 L2	Mettu19	2.17	7	2.17	0.08	61.81	10	17
Y2 L3	Asosa19	3.03	2	3.03	1.15	8.03	2	4
Y2 L4	Shire19	3.24	1	3.24	0.47	22.19	7	8
Y2 L5	Gonder19	1.87	10	1.87	-0.07	48.11	9	19
Y2 L6	Jinka 19	2.38	6	2.38	-0.37	15.48	4	10
Y2 L7	Tepi19	2.13	9	2.13	0.02	293.91	11	20

ASV, AMMI stability value; IPCA1 and IPCA2 are the first and second interaction principal component axes; respectively and GSI, Genotype selection index; RYi, the sum of the rank of the genotype grain yield and RASVi , the rank of the genotype ASV (RASVi).

### 3.4 AMMI biplot analysis

**Figure 2:**
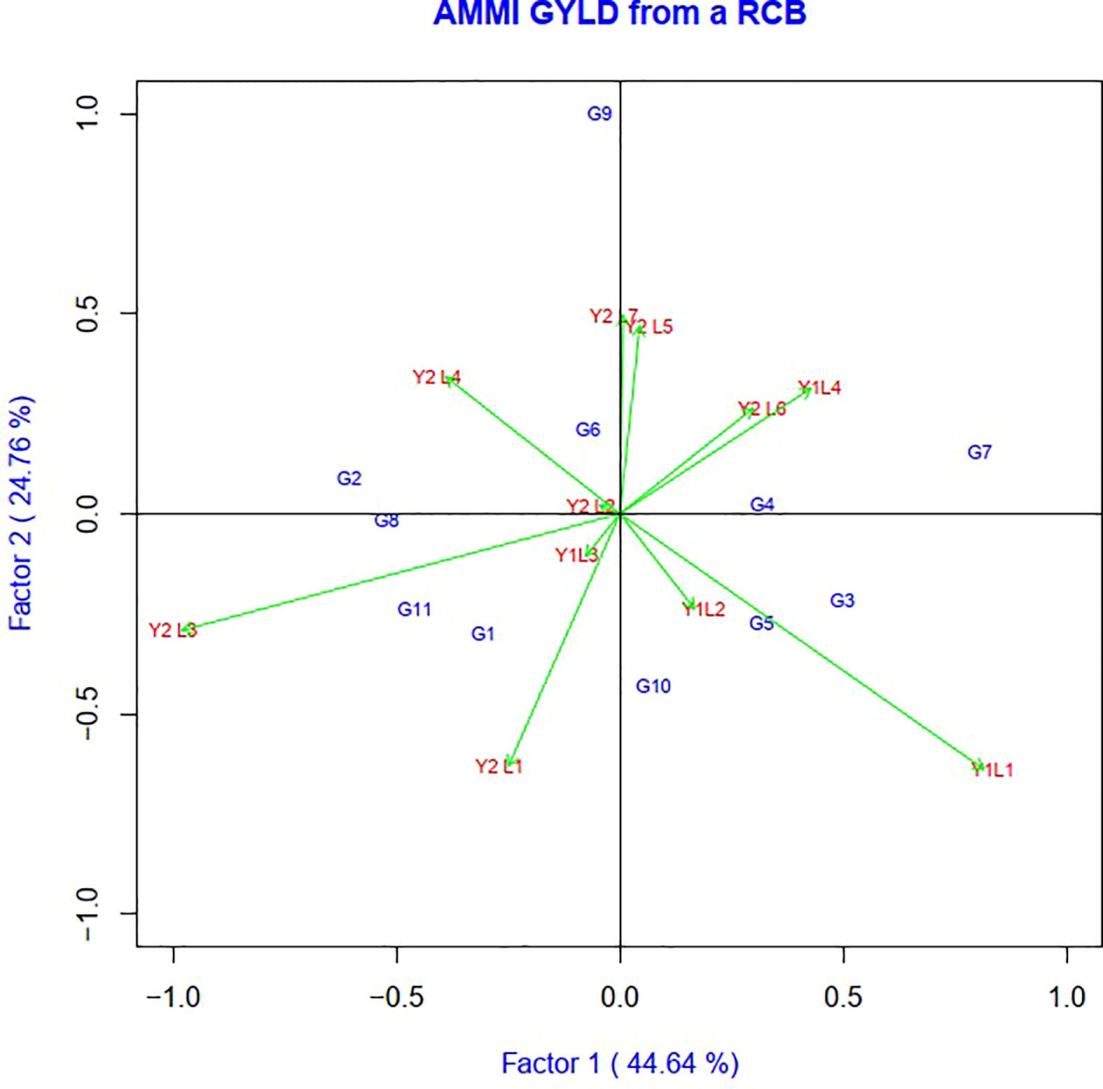
The biplot ‘AMMI 2’ illustrated the first two principal components (PC1 and PC2) effects of genotype plus GE interaction effect of 11 soybean genotypes under two years (11environemnets) for grain yield per hectare. The biplots were created based on Centering = 0, SVP = 2, Scaling = 0.

The AMMI model analysis was used in this study to provide a substantial and informative summary of GEIs, as well as to examine the correlations and differences in genotype performance across diverse testing conditions. In the AMMI 1 biplot, the usual interpretation of the biplot is that the displacements along the abscissa indicate differences in main (additive) effects, whereas displacements along the ordinate indicate differences in interaction effects. The first two principal components, IPCA1 and IPCA2, explained 69.37% of the total GEI variation (**Figure 2**). In terms of the grain yield feature, G8 > G2 > G1 > G11 and G5 showed the minimum interplay between genotypes and environments.

**Figure 3:**
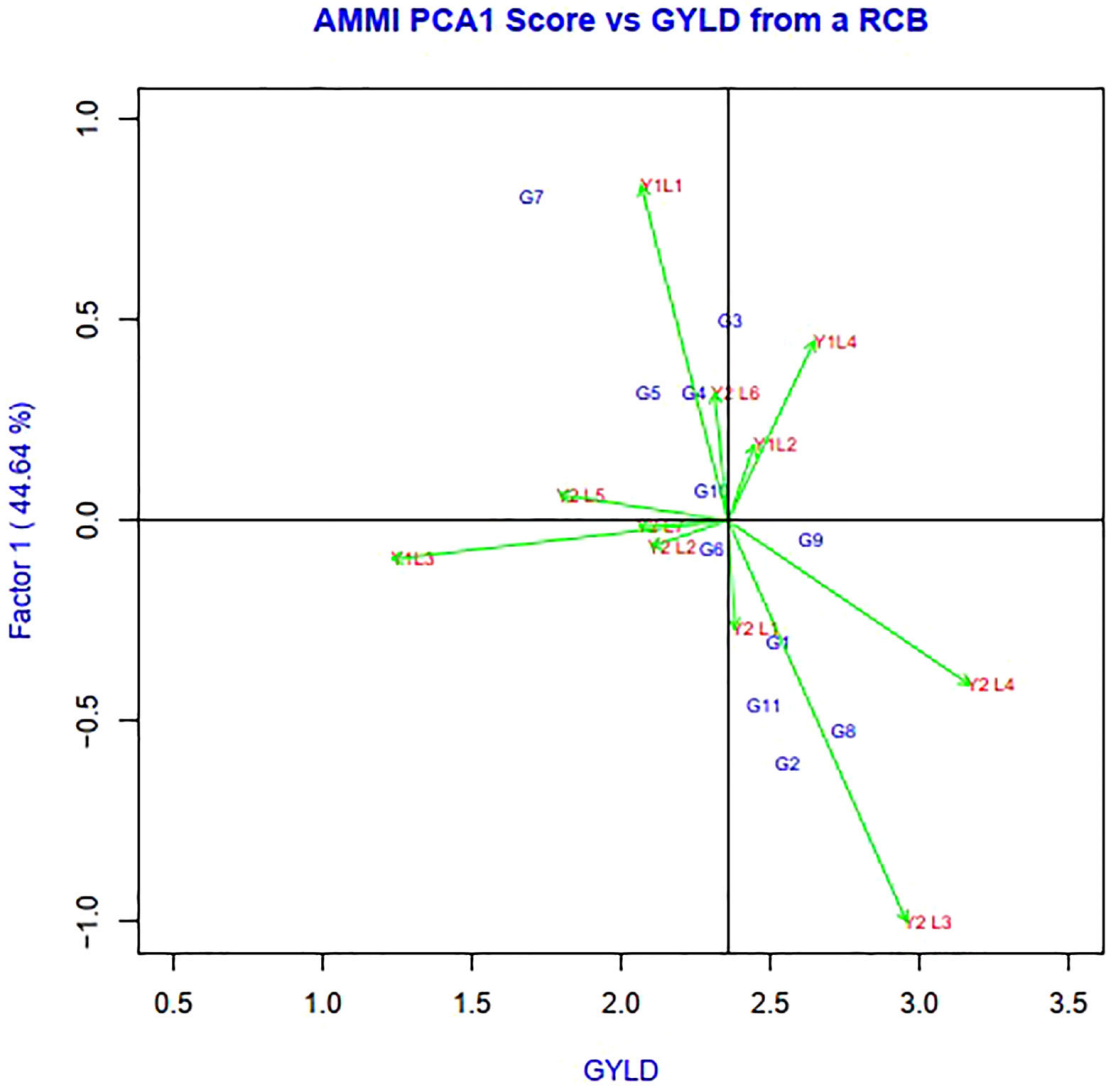
The biplot ‘AMMI 1’ illustrated the trait main effect and first interaction principal component (IPC1) effects of both genotype and environment of 11 soybean genotypes under two years (11 environments) for grain yield per hectare. The biplots were created based on Centering = 0, SVP = 2, Scaling = 0.

Genotypes having a zero IPCA 1 score are less influenced by the environment and better adapted to all environments. Since the IPCA 1 scores of varieties G2, G6, G8, G4 and G9 were close to zero, they were the most adapted and stable genotypes across these environments (**Figure 3**). However, the mean yield of genotype G8 was higher than the remaining genotypes, so it is preferable since it had a mean yield above average, while the other two genotypes, G6 and G4, had a mean below average. In summary, an adapted and stable variety might not be the highest yielding.

The environmental score is connected to the origin by side lines. Short arrow does not provide powerful interactive power. The long arrow has strong interactions. Genotypes near the coordinates represent general adaptations, and further genotypes represent more specific adaptations to the environment (Gauch and Zobel, 1996). The scatter plot of grain yield vs. IPCA1 (**Figure 3**) illustrates that the superior genotype had a higher agricultural yield and IPCA1, which is shown on the vertical axis, had a minimum value and was near zero. The superior genotypes were G8< G2< G9. Whereas, it is located on the right side of the graph and close to zero in terms of the IPCA1 axis. The most unstable genotypes and the lowest grain yield among the genotypes belonged to G5 and G10, G7, G4, G6, and G8 had the least GEI. Y1L1, Y2L1, Y2L7, and Y2L5 were the unstable environments.

## 4 GGE biplot analysis

**Figure 4:**
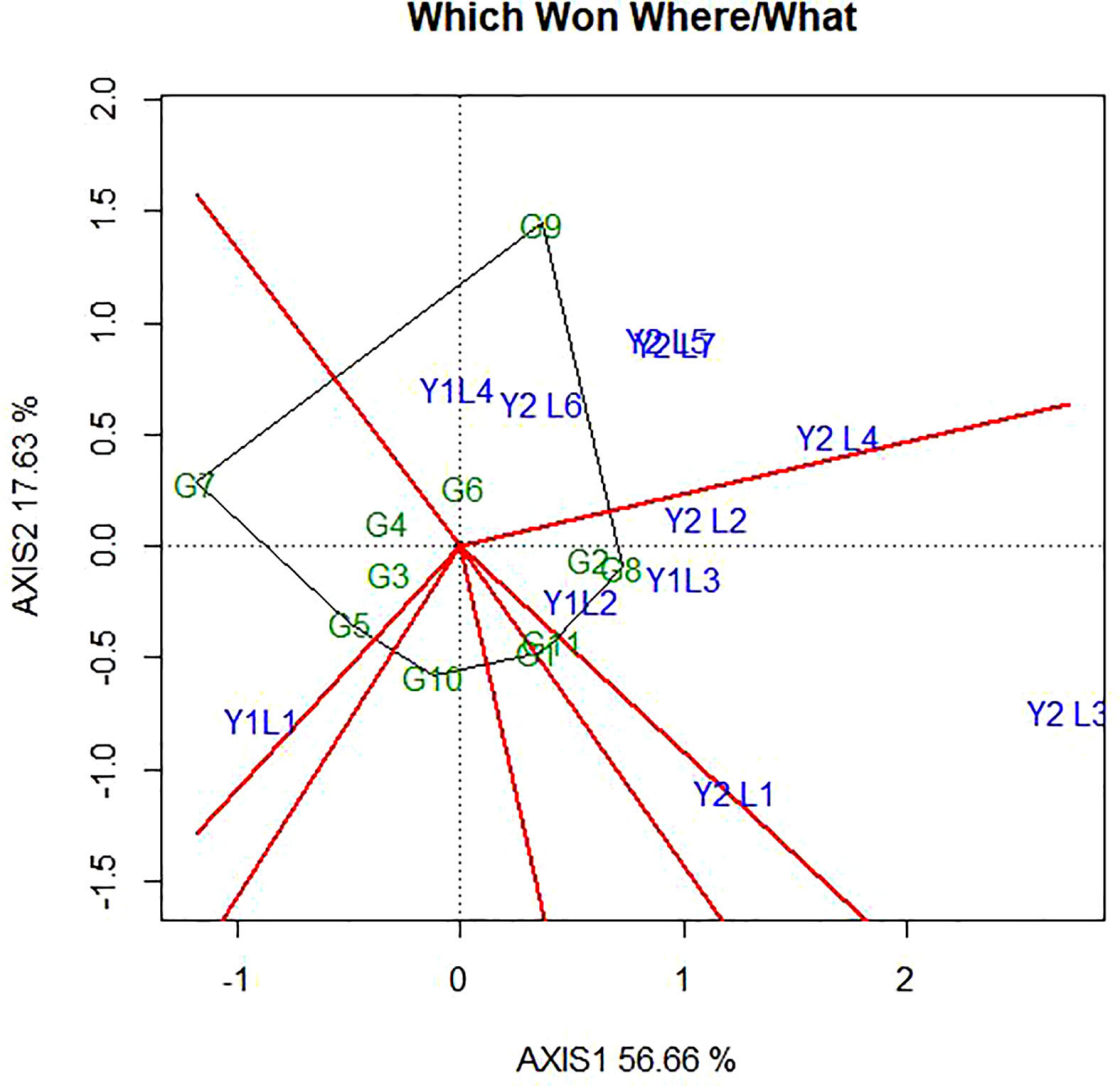
”Which-won where” pattern of GGE biplot polygon view displaying the genotype main effect plus GEI effect of 11 soybean genotypes under two years (11 environments) for grain yield per hectare. The biplots were based on Centering =0, SVP=2, Scaling=2.

The genotype + genotype × environment (GGE) biplot analysis result of the eleven soybean genotypes evaluated across environments with respect to yield. The GGE biplots explained 74.29% of the total variation distributed as 56.69% and 17.62% of sum of squares between principal component PC1 and PC2, respectively (**Figure 4**).

### 4.1 Which-won-where polygon view of GGE biplot

The polygon is formed by connecting the signs of the genotypes that are farthest away from the biplot origin, such that all other genotypes are contained in the polygon. In this case, the polygon connects all the farthest genotypes and perpendicular lines divide the polygon into sectors. Sectors help to visualize the mega-environments. This means that winning genotypes for each sector are placed at the vertex. The pattern on the environment in the biplot suggests that the existence of seven sector and five different mega-environments (**Figure 4**). The vertex genotype of each sector is the one that gave the highest yield for the environments which fall within that sector. The vertex genotypes in this study were G2, G11, G7, G8, G10, G1 and G9. According to Yan and Tinker (2006), the vertex genotypes were the most responsive genotypes, as they have the longest distance from the origin in their direction. From this figure, G9 was the best performer at Y2L5, Y2L7, Y1L1, Y2L6 and Y2L4 on the first environment; the second environment containing the higher yielding environment Y2L2, Y1L3, Y1L2 and Y2L3 with a winner genotypes G8 and G2. The third environment include Y2L1 with a vertex genotype G11 and G1 while the fourth environment include only Y1L1 with the winner genotype G7. From the figure 4, G10 was on the vertex with no environment. However, genotypes within the polygon, particularly those located near the biplot origin were less responsive than the genotypes on the vertices and the ideal genotype would be one closest to the origin.

### 4.2 Relationship among environments

**Figure 5:**
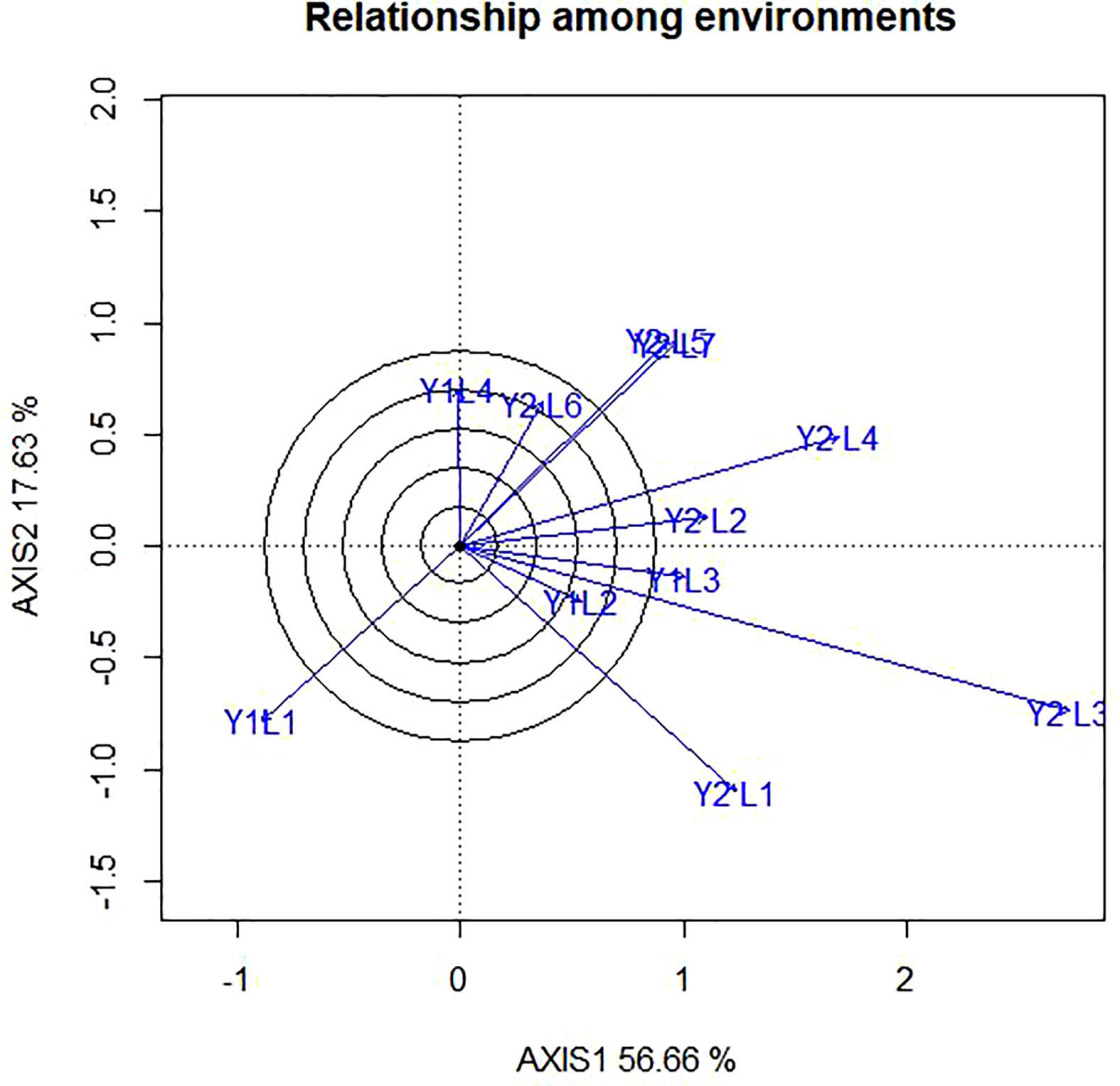
The vector view of GGE biplot showing the relationship among environment (tested environment with the ideal environment) of 11 soybean genotypes two years (11 environments) for grain yield per hectare. The biplots were created based on Centering = 0, SVP = 2, Scaling = 0.

In the present study, the relationships among the test environments are presented in **Figure 5**. The lowest angle was observed between Y2L5 and Y2L5, Y1L3 and Y2L3 followed by those between Y1L2 and Y2L1indicating the existence of high correlation between them. This shows that genotypes performing best at Y2L5 can repeat the same performance at Y2L5, and vice versa. The angles between Y2L1and Y2L5 on the other hand, were closer to 90° showing that they have no correlation or indicating that each environment has independent genotypic performance. Furthermore, the angle between Y2L1 and Y2L6 is greater than 90^0^ showing that they have negative correlations. Hence, genotypes performing best at Y2L1 does not repeat the same performance at Y2L6, and vice versa. Based on the representativeness and discriminating ability of the study environment, Yan et al. (2007), classified the environment into three major types. Type 1 environments are characterized by short vectors, providing little or no information genotypes; thus, they are inappropriate as test environments. Type 2 environments are characterized by long vectors, forming smaller angles with the AEC abscissa, and are useful for selecting superior genotypes. Type 3 environments with long vectors form large angles with the AEC abscissa; therefore, they are inappropriate for the selection of superior genotypes. Despite their limitation, Type 3 environments can be useful in culling unstable genotype. The eleven test environments in the present study were categorized into three based on the relationship among environments. Thus, category-I contained of Y2L5, Y2L7, Y1L1, Y2L6, Y2L3, Y2L4 and Y2L2; category-II comprised Y2L3, Y1L1 and Y2L1, whereas the category-III consisted of Y1L1 only. The categorizing was based on relationship among environments is in line with the environmental categorizing of the polygon view.

### 4.3 Discriminativeness vs representativeness

**Figure 6:**
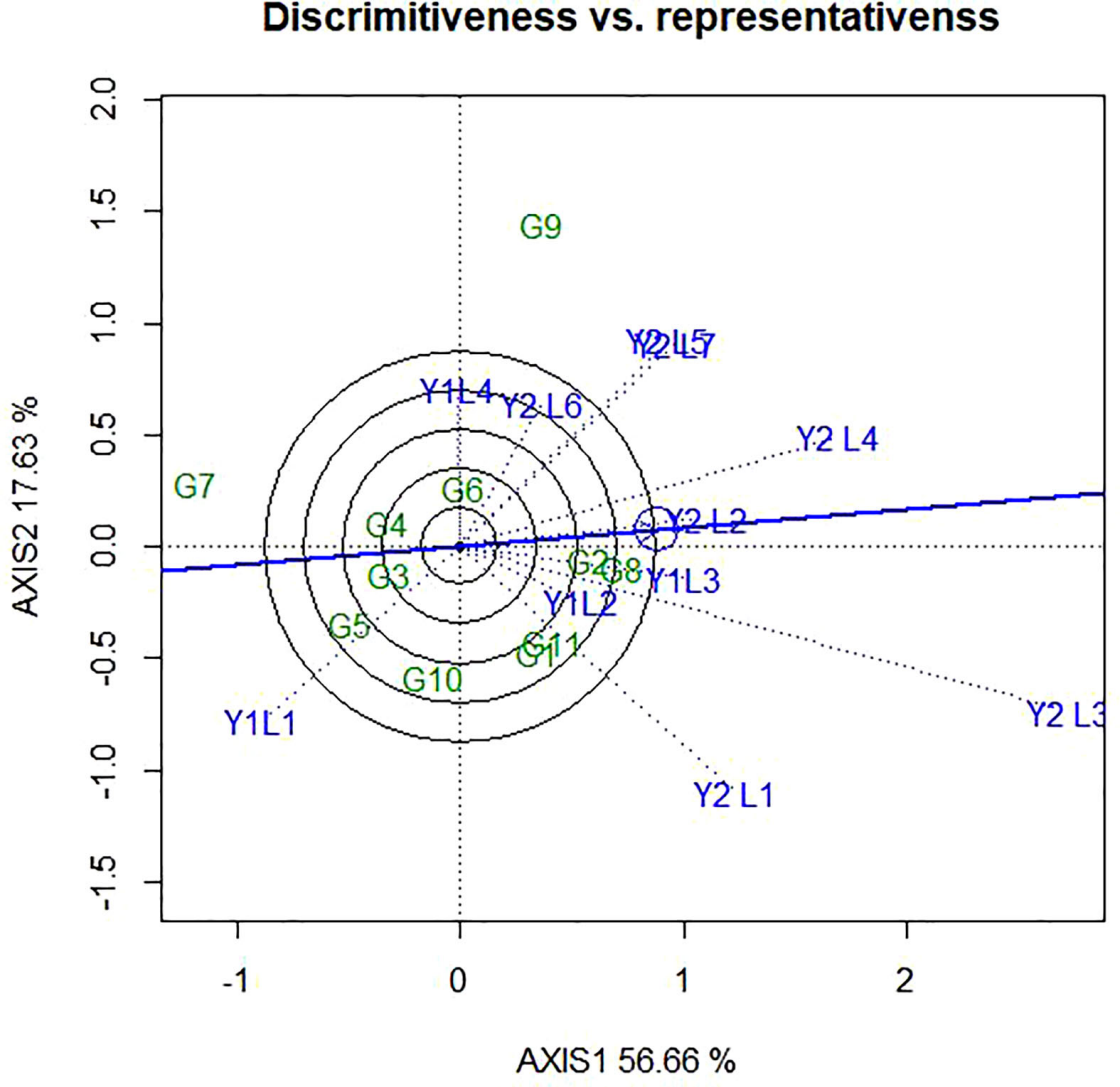
The GGE biplot ‘Discriminativeness vs. Representativeness’ pattern for genotype comparison with ideal genotype showing G + G × E interaction effect of 11 soybean genotypes under two years (11 environments) for grain yield per hectare. The biplots were created based on Centering = 0, SVP = 2, Scaling = 0.

It is known that the GGE biplot is also useful to assess how much a test environment is capable of generating unique information about the differences among genotypes and how representative the mega-environment is. In this study, based on the length of vector the different environments (**Figure 6**), Y2L3, Y2L1, Y1L1, Y2L4, Y2L5 and Y2L7 had the longest vector length and, therefore, are the most discriminating environments in the present study. Similarly, Y1L2, Y2L2 and Y1L3 had the shortest vector length which indicates that they are the least discriminating of all the test environments.

### 4.4 Genotype ranking based on their mean performance and stability

**Figure 7:**
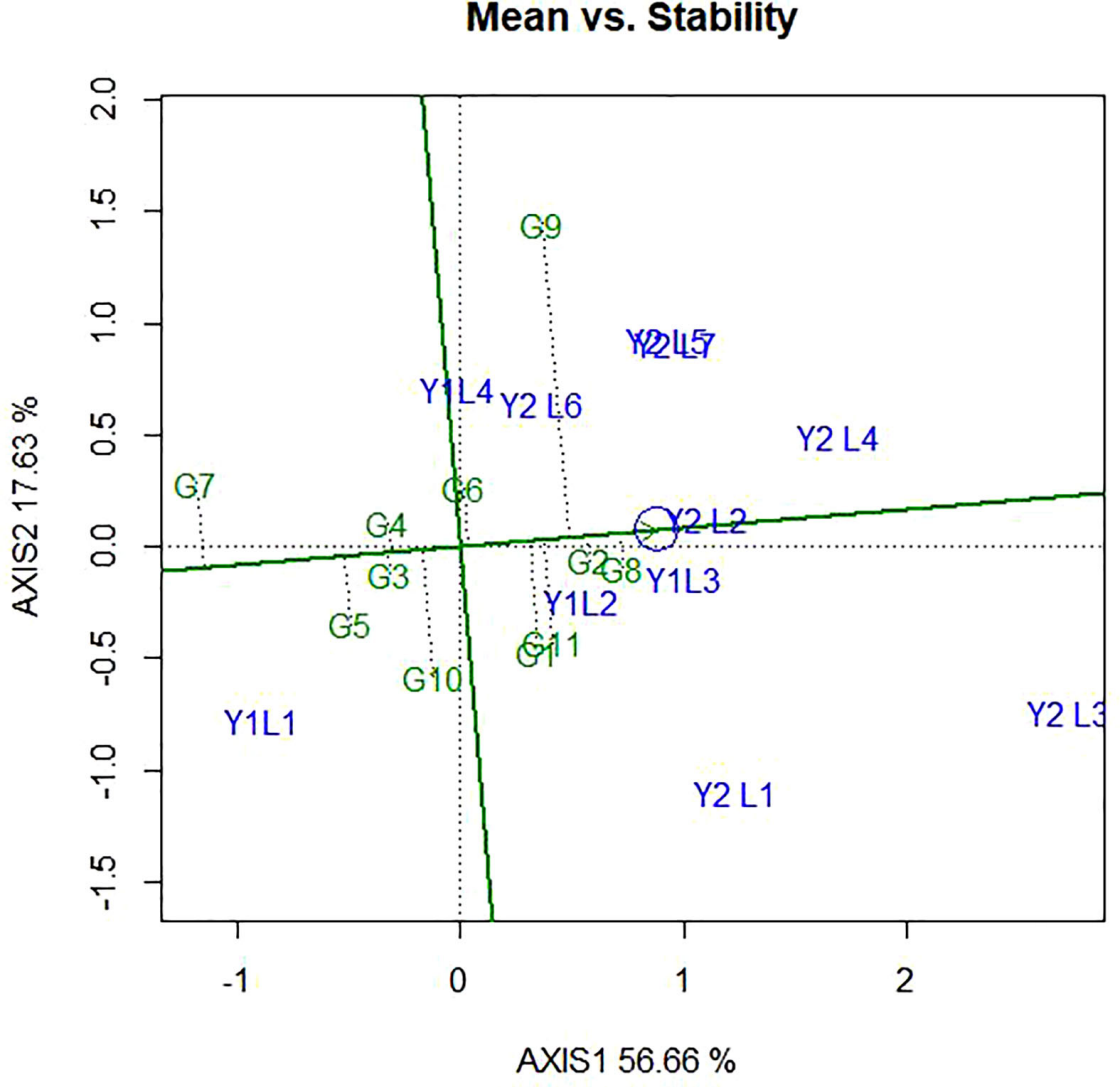
The ‘‘Mean vs. stability’’ pattern of GGE biplot illustrating interaction effect of 11 soybean genotypes under two years (11environemnets) for grain yield per hectare. The biplots were created based on Centering = 0, SVP = 2, Scaling = 0.

The ranking of the genotypes based on their mean performance and stability presented in **Figure 7**. It has been established that if the PC1 of a GGE biplot approximates the genotype main effects, PC2 must approximate the GE effects associated with each genotype, which is a measure of instability (Yan et al., 2000; Yan, 2001). The line passing through the biplot origin and the average environment indicated by a circle is called the average environment coordinate (AEC) axis, which is defined by the average PC1 and PC2 scores of all the environments. By using the average principal components in all environments, the AEC method was employed to evaluate the yield stability of genotypes. In our study, the “mean vs. stability” pattern of GGE biplot revealed 74.29% for yield per hectare of G + G E variation (**Figure 7**). A line drawn through the average environment and the biplot origin, having one direction pointed to a greater genotype main effect. Moving in either direction away from AEC ordinate and from the biplot origin indicates the greater GEI effect and reduced stability. The genotypes with below-average means and those with above-average means are divided by the AEC ordinate. As a result, genotypes G8, G11, G1, G2, and G9 showed yield performances that were higher than the average yield in this study (**Figure 7**). The genotypes G7, G10, G5, G4 and G3 had lesser yielding performance in comparison to the mean, while genotypes G8, G2 and G4 are more adapted and stable. This is because the genotype on the right of the ordinate line had yield less than the average mean yield. The least stable genotypes are G9 and G10, on the other hand. Stability can be identified based on concentric circles and also ideal genotypes are on the centre of concentric circles i.e., high mean and stable. Beside this, good genotypes are close to ideal genotypes.

### 4.5 Evaluation of genotypes relative to ideal genotypes

**Figure 8:**
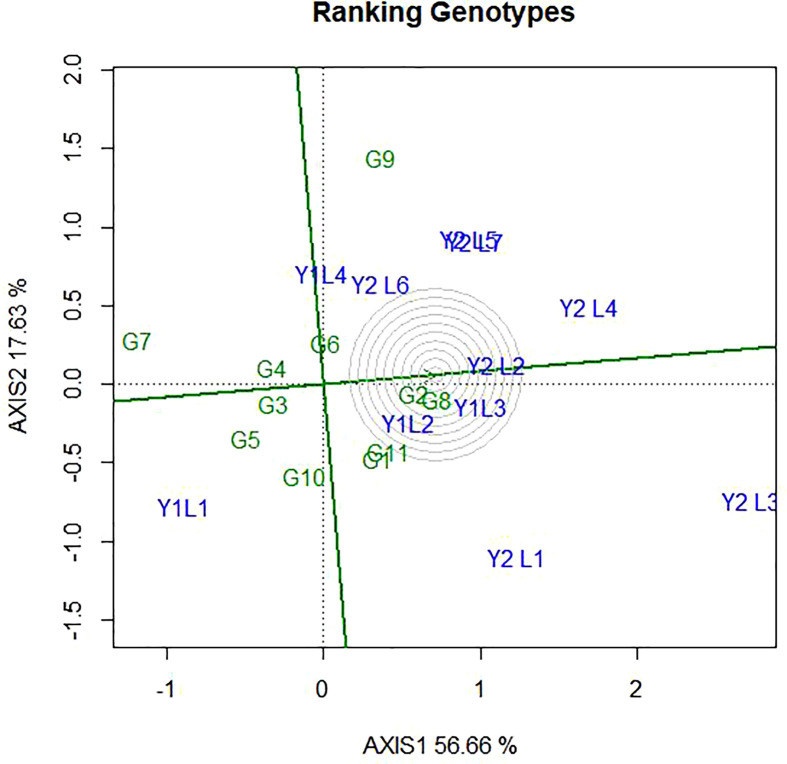
The GGE biplot ‘Genotype ranking’ pattern for environment comparison with ideal environment showing G + G × E interaction effect of 11 soybean genotypes under two years (11environemnets) for grain yield per hectare. The biplots were created based on Centering = 0, SVP = 2, Scaling = 0.

The ideal genotype is the one that with the highest mean performance and absolutely stable (Yan and Kang, 2002). This is assumed to be in the centre of the concentric circles is an ideal genotype across the tested environment. It is more desirable for a genotype to be located closer to the ideal genotype. Hence, the GGE biplots (**Figure 8**) shows that G8 and G2 were ideal in terms of higher-yielding ability and stability as compared to the other genotypes. While genotypes G7, G5, G9, G10 and G5, were unfavourable since there are too far from the ideal genotypes. Some of the environments located close to the ideal genotypes (Y1L2, Y1L3, and Y2L2) and most (Y2L3, Y1L1, Y2L1, and Y2L4) are located far from the ideal variety.

### 4.6 Evaluation of environments relative to ideal environments

**Figure 9:**
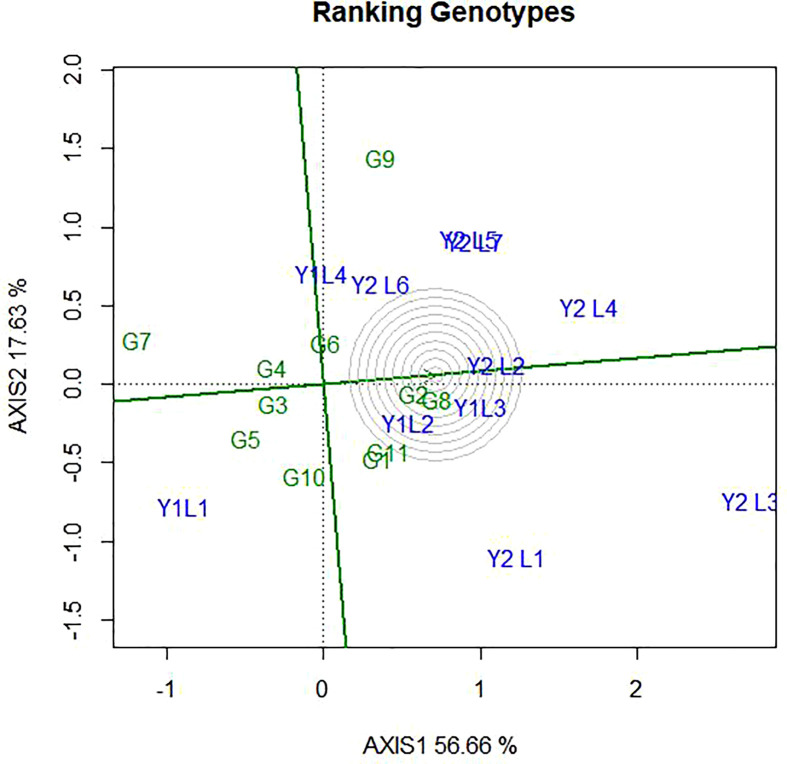
The GGE biplot ‘Environment ranking’ pattern for environment comparison with ideal environment showing G + G × E interaction effect of 11soybean genotypes under two years (11environemnets) for grain yield per hectare. The biplots were created based on Centering = 0, SVP = 2, Scaling = 0.

Ideal environments had the longest vector with small IPCA, which fell into the centre of concentric circles. Hence, from this study Y2L4 and Y2L3 are ideal environment. Ideal environment is the most representative of the overall environments and the most powerful to discriminate genotypes. Likewise, Y2L2 and Y1L3 were closer to the ideal environment and considered as second powerful to discriminate genotypes. On the other hand, environments Y1L1 and Y1L4 were found far from the ideal environment and considered as less powerful to discriminate genotypes (**Figure 9**).

## 5 Discussions

Ethiopia still imports processed soybean products such as soya edible oil and other oil crops like Sesame and Niger seed. Soybeans generated $376 million (USDA, 2021) and around 1.4 million farmers produced oilseed crops in the country (CSA, 2020). The mean square of environments, genotypes, and genotypes by environments interaction showed very highly significant differences (p ≤ 0.001) for yield per hectare. This is an indication that the response of soybean genotypes is highly dependent on the site where the genotypes are grown and annual variation of temperature and rainfall. The results also indicate that environmental factors have a significant impact on the performance of different soybean genotypes, indicating that different soybean genotypes should be tested over time and in different locations. The highest grain yield in tone was recorded at Y1L4 (3.2), followed by Y2L3 (3.0) and lastly, Y2L4 (2.7). The influence of genotype, environment, and GEI in the soybean for different traits has been reported by (Gurmu et al., 2009; Agoyi et al., 2017; Mwiinga et al., 2020).

GGE biplot can be graphically detect the genotype by environment interaction pattern, identify winning genotype, and delineating mega environments among the testing environments (Yan et al., 2007). So, this potential tool has been employed to analyze the multienvironment data of grain soybean trials (Mwiinga et al., 2020). In our study, environment contributed 49.42% of the variation in the data, while the contribution of genotype and their interaction with environment was around 33.91%. Gauch and Zobel (1997) reported that normally in MET data, the environment accounts for about 80% of the total variation. Zamalotshwa et al. (2020) found that 89.65 and 89.06% of the difference in Glu and Glut was explained by the environment in soybean-MET data. Agoyi et al. (2017) reported a similar trend for soybean dry weight of nodules at 83.79% and the percentage of effective nodules was 12.98%. However, Agoyi et al. (2017) reported a slightly lower (12.98%) contribution of environment to the total variation in soybean yield number of nodules from Uganda. In a two-year study of eleven diverse lines across seven environments, Mwiinga et al. (2020) attributed 21.04% of overall variation to environment, 31.59% to genotype main effect, and 47.36% to GEI in a two-year study of eleven diverse lines. In our study, GEI explained a higher proportion of the variation than genotype alone. The higher proportion of GEI as compared to genotype is indicative of the possible existence of different mega-environments in testing environments (Yan, 2002; Yan and Kang, 2002). This could be true not only in Ethiopia, but also in other soya-growing regions. But in the presence of the GEI effect could complicate the selection process of superior genotypes and also may reduce the selection efficiency in breeding programs (Gauch, 2006; Kumar et al., 2014). As a result, when breeding soybeans in their individual environments, soybean breeders must consider this fact.

### 5.1 Mean performance and comparison of genotypes

Plant height, number of seeds per plant, number of pods per plant, and weight of 100 seeds are important agriculture for soybean producers to develop excellent varieties of soybean. It will be the standard. A genotype that is adapted and stable to yield in a variety of environments, on the other hand, is highly valued by researchers in a breeding program to limit the risk of yield loss due to climatic circumstances. The results also demonstrated that the control Nyala (2.35 t ha^-1^) gave the sixth and unreleased genotypes, which come from the modified single descent method from the soybean breeding, namely JM-CLK/CRFD-15-SD (2.84 t ha^-1^), and the remaining three genotypes through direct introduction from various sources, namely 5002T (2.73 t ha^-1^) and PI471904 (2.64 t ha^-1^) and SCS-1 (2.57 t ha^-1^ were among the top performers with respect to yield and stability. The genotype Nyala, which was released in 2014, was among the top five ranked genotypes. When genotype performance varies in a variety of environments, Gurmu et al. (2009) and Asrat et al. (2009), recommends studying GEI followed by stability analysis. Similarly, studying on seven soybean promising lines and two check varieties, on eight locations of soybean production, TGx-1835-10F and TGx-1876-4E exhibit higher stability than other genotypes (Asrat et al. 2009). In evaluating the performance of five soybean genotypes, Al-Assily et al. (2002) found that three cultivars consistently produced mean yields that were higher than the trial mean. In a similar regard, Cucolotto et al. (2007) discovered that out of 30 cultivars, four had good adaptation and stability. In contrast to our findings, Asrat et al. (2009) did not report any genotype with widespread adaption.

### 5.2 The AMMI model analysis

The application of AMMI model for partitioning of GEI showed that the first four (IPCA1-4) multiplicative terms of AMMI were significant (**Table 6**) and this implies that the interaction of 11 genotypes of soybean with eleven environments was predicted by the first four principal components of genotypes and environments, which is in agreement with the recommendation of Behrouz et al. (2018) and Goa et al. (2022). This is also following the results of Mwiinga et al. (2020) and Mushoriwa et al. (2022), whereas much as the first five and six IPCAs were significant respectively. However, this contradicted with the findings of Zobel et al. (1988) and Abiriga et al. (2020), which recommended that the most accurate model for AMMI, can be predicted using the first two IPCAs.

**Table 6:** Grain yield AMMI investigation of variance across seven locations in eleven environments.

Sources of Variations	df	SS	MS	Total Variation (%)	GEI Explained (%)	GEI Cumulative (%)
Block, Env.	33	15.54	0.471			
Genotypes, G	10	36.61	3.661***	16.18		
Environments, E	10	126.65	12.665***	49.42	49.42	
Interactions, GEI	100	83.56	0.836***	33.91		
IPCA 1	19	37.29	1.963***		47.74	47.74
IPCA 2	17	20.67	1.216***		26.62	74.36
IPCA 3	15	13.78	0.919***		17.88	92.23
IPCA 4	13	5.93	0.456*		7.77	100.00
Error	330	82.15	0.249			
Total	483	344.51	0.713			

GEI, Genotype by Environment interaction; df, degree of freedom; SS, Sum Square; MS, Mean Square; IPCA, Interaction principal component axis.

***Significant at P < 0.001, *Significant at P < 0.05.

Environments accounted for the largest share (49.42%) of the overall treatment sum of squares in this study, followed by GEI (33.91%) and genotypes (16.18%), which supports the necessity for multi-environmental trials conducted throughout several seasons in this nation. The environment was revealed to be the main source of variance in additional studies by Gurmu et al. (2009); Rakshit et al. (2012); Mushoriwa et al. (2022), and Vaezi et al. (2018). In contrast, studies by Mulugeta et al. (2013), Mwiinga et al. (2020), and Bhartiya et al. (2017) likewise found that the GEI contributed more to overall variation, with values of 60%, 47.36%, and 41%, respectively.

AMMI 1 identified the genotypes as the most stable and high producing genotypes based on how well they performed in various situations (G8, G2, and G9). The highly interacting environment (Y2L3) and genotypes (G9 and G7) were found in the AMMI 2 biplot. Biplot has also been used to illustrate how precisely genotypes adapt to the right environment. The genotype G9 benefited more from the use of Y2L7.The genotype G3 interacted favorably with Y1L1, while the genotype G7 expressed the high yielding potential in Y1L4. Similar positive interactions were seen between the genotypes G5, G6, G1, G6, G11, G2, G6, and G4 and the locations Y1L2, Y1L3, Y2L1, Y2L2, Y2L3, Y2L4, and Y2L5.

According to similar approaches applied, Mwiinga et al. (2020), the genotypes and the environments average was 2.4 t ha^−1^.The precise adaptation of genotypes to the appropriate environment was also visualized using biplots (Samyuktha et al., 2020).

The AMMI stability values (ASV), IPCA and GSI scores were used to classify the genotypes according to stability. With consideration, according to ASV, the genotypes close to 0 (G6, G1, G10 and G5) were highly adapted and stable, whereas the genotypes having high value (G8, G7, G2, and G4) were found to be highly unstable. From the ASV, both the top and the lowest yielder *viz*., G8 and G7 categorized under unstable genotypes. Based on GSI, the following genotypes *viz*., G9 (PI471904), G1 (5002T), and G11(Nyala) were identified as stable and high yielding across all the test environments under study. In the study, Mushoriwa et al. (2022), identified both the IPCA scores and GSI identified genotype G25 was the most superior followed by genotype G1 while genotype G16 performed the least among the 42 studied genotypes. At the same time these authors further explained that IPCA and GSI stability parameters could be used for simultaneously selection for high yield and stability. Therefore, the two stability parameters could be used for simultaneously selection for high yield and stability. From the results of Mwiinga et al. (2020) shows that, the same results and reason were obtained in studying on twenty elite soybean lines and five commercial checks across six environments across four Southern Africa countries, in six environments. In addition, Mushoriwa et al. (2022), considering the two analytical methods (AMMI and GSI), two genotypes (Sovreign and SC Siesta) were among the best and thus could be recommended for cultivation across the three countries and utilization as breeding stocks in programs that aim to improve both stability and productivity of soybean.

### 5.3 GGE biplot analysis

In the GGE bi-plot analysis, the variation 74.29% for the first two principal components was higher than the ideal limit (66%) (Yan et al., 2000). Similarly, this result is lower than that obtained by Arega et al. (2020) (93.59%), Amira et al. (2013) (86.6%); Carvalho et al. (2021) (80%); Mulugeta et al. (2013) (63.4%), Krisnawati and Adie (2018a) (60.87%) and Bhartiya et al. (2017) (74.40%) in contrary to that found by Kocaturk et al. (2019) (51.90%).

*GGE biplot (‘Which-won-where’ pattern)* - According to Yan and Tinker (2006), the vertex genotypes were the most responsive genotypes, as they have the longest distance from the origin in their direction. In this study since there are five mega environments, but from the Yan et al. (2007), infer that the repeatable environment grouping is necessary, but not sufficient, for declaring different mega-environments. The necessary and sufficient condition for mega-environment division is a repeatable which-won-where pattern rather than merely a repeatable environment-grouping pattern (Yan and Rajcan, 2002; Yan and Kang, 2002). This indicates that genotypes in vertex without environment performed poorly in all the sites (Alake and Ariyo, 2012). Similarly, Mulugeta et al. (2013) found that from the polygon view of this biplot, test environments and genotypes fell into three and four sectors, respectively, with three sectors having no test environment.

### 5.4 Relationship among environments

GGE biplot analysis helped us to understand the utility of different sites in terms of relative discrimination between genotypes and the relationship between them for different traits. The grouping based on relationship among environments is in line with the environmental grouping of the polygon view. According to Yang et al. (2009), an ideal environment should have a high PC1 score and zero scores for PC2. Similar results to our findings were stated by Zhang et al. (2006) and Bhartiya et al. (2017). In the study by Mulugeta et al. (2013), identified three environments namely “Dibate_2008”, “Bullen_2007” and “Pawe_2007” were the most discriminating of the genotypes whereas “Dibate_2007” provided very little information about genotypic differences in the north western regions of Ethiopia.

### 5.5 Discriminativeness vs representativeness

According to Yan et al. (2007), described the discriminating power vs. representativeness view of the GGE biplot is an effective tool for test-environment evaluation, which can lead to the identification of a minimum set of discriminating and representative test environments. Test-environment evaluation has not been a research topic in AMMI analysis. The Y1L2, Y2L2 and Y1L3 had the shortest vector length which indicates that they are the least discriminating of all the test environments. Hashim et al. (2021), reported one environment is suitable for genotype selection considering yield per hectare among the tested four environments.

### 5.6 Genotype ranking based on their mean performance and stability

The present study showed that there were a number of genotypes that performed better than the control Nyala (G11) the most farmer-preferred and high yielding varieties in Ethiopia. This study also showed that JM-CLK/CRFD-15-SD(G8) derived through across modified single seed decent (MSSD) plant selection Clarck -63K and Crowford had the highest yield level compared to all the other genotypes. Stability can be identified based on concentric circles and also ideal genotypes are on the centre of concentric circles.

### 5.7 Evaluation of genotypes relative to ideal genotypes

According to Yan et al. (2007), genotype evaluation is meaningful only for a specific mega-environment, and an ideal genotype should have both high mean performance and high stability within a mega-environment. The ideal genotype is the one that with the highest mean performance and absolutely stable (Yan and Kang, 2002). According to Yan and Tinker (2006), the most responsive genotypes were located at the vertexes of the polygon, since they have the longest distance from the origin in their direction. Oladosu et al. (2017) finds out similar results observation.

### 5.8 Evaluation of environments relative to ideal environments

According to the biplot graph, the biplot accounted for 56.66% (PC1) and 17.63% (PC2) of the variability in grain yield of G+GEI across the studied environment. Mushoriwa et al. (2022) found that the majority of the settings had the fewest interaction effects, indicating that they were perfect for evaluation and selection since the genotypes’ performance could be consistent in them. In this study, eleven test environments in were categorized into three based on the relationship among environments and thus, category-I contained of Y2L5, Y2L7, Y1L1, Y2L6, Y2L3, Y2L4 and Y2L2; category -II comprised Y2L3, Y1L1 and Y2L1, whereas the category -III consisted of Y1L1 only. In a similar study by Khan et al. (2021), the tested environments were categorized in to three groups for different agronomic traits. In a similar study, Arega et al. (2020) identified the test location (Billo and Gute) as the most discriminating environment, while Bako and Uke were identified as the least discriminating testing environments. Chewaka was identified as the least discriminating environment among the testing environments. “Bullen_2007”, “Dibate_2007”, and “Manbuk_2008” were found to be more representative environments for soybean regional trials by Mulugeta et al. (2013).

## 6 Conclusion

This research evaluated the stability and adaptability of soybean genotypes based on combined ANOVA and, AMMI, GGE biplot analysis. Analysis of variance for every location and combined over locations showed significant differences among genotypes, environments and GEI for grain yield, as well as most of the yield-related traits of soybean. According to AMMI and GGE analyses to generate adapted, stable and high-yielding soybean genotypes across several environments would be beneficial. Five different mega-environments and seven different sectors are indicated by the biplot’s environment pattern. The vertex genotypes used in this study were G2, G11, G7, G8, G10, G1, and G9. In the first environment’s Y2L5, Y2L7, Y1L1, Y2L6, and Y2L4 circumstances, the genotype G9 performed best. In the second environment’s Y2L2, Y1L3, Y1L2, and Y2L3 conditions, the winning genotypes G8 and G2 had greater yields. The third environment also comprises Y2L1 with vertex genotypes G11 and G1, but the fourth environment only has Y1L1 with the winning genotype G7. Unusually, the genotype G10 vertex lacked an environment.

The yields of genotypes G8 and G1 ranged from 2.85 to 2.75 t ha^-1^ and were significantly greater than those of other genotypes and the control variety. The results from the four distinct stability statistics AMMI biplot (G8, G2, G1, G11, ASV: G1, G11; GSI; G9, G1, G11 and GGE: G2, G8, G9 are taken into account together with the genotypes’ grand mean. The genotypes JM-CLK/CRFD-15-SD (G8) and 5002T (G1), which rank among the best and have the highest seed output, are suitable for hybridization as a parent and commercial production. The highly high grain output may be due to genotype variations in relative performance across environments and their changing sensitivity to a variety of abiotic variables seen in a typical Ethiopian soybean growing environment.

## Data availability statement

The raw data supporting the conclusions of this article willbe made available by the authors, without undue reservation.

## Author contributions

MH took part in the conception and design of the study, data collection, paper writing, statistical analysis, and interpretation of the data, as well as article revision for important intellectual components. The final text has been reviewed and approved by the author.

## Funding

Ethiopian Institute of Agricultural Research (EIAR), Ethiopia.

## Acknowledgments

The authors appreciate the financing provided by the Soybean Innovation Laboratory (SIL), as well as the experimental lines and other resources provided by EIAR. In addition, the Ethiopian Institute of Agricultural Research [EIAR-Soybean (1-2-15)] generously funded this research.

## Conflict of interest

The author declares that the research was conducted in the absence of any commercial or financial relationships that could be construed as a potential conflict of interest.

## Publisher’s note

All claims expressed in this article are solely those of the authors and do not necessarily represent those of their affiliated organizations, or those of the publisher, the editors and the reviewers. Any product that may be evaluated in this article, or claim that may be made by its manufacturer, is not guaranteed or endorsed by the publisher.
