# Supplementary material for: Adaptability and stability for soybean yield by AMMI and GGE models in Ethiopia
Source: Front Plant Sci. 2022 Nov 23;13:950992. doi: 10.3389/fpls.2022.950992 (PMC9727298; doi:10.3389/fpls.2022.950992)
Supplement: Supplementary file 1 [file Table_1.docx]

Table 1. Barely used genotypes used for the study

| **Accession No** | **Region** | **Zone** | **Woreda** | **Local Vernacular Name** | **Latitude** | **Longitude** | **Altitude** | **Sample** | **Nature of Samples** | **Soil PH** |
| --- | --- | --- | --- | --- | --- | --- | --- | --- | --- | --- |
| 3841 | SNNP | SEMEN OMO | SODO ZURIA | Banga | 06-53-00-N | 37-50-00-E | 2000 | Population / Mixture | Spikes | 6 |
| 3840 | SNNP | SEMEN OMO | SODO ZURIA | Gebs | 06-54-00-N | 37-48-00-E | 2180 | Population / Mixture | Spikes | 6 |
| 3838 | SNNP | SEMEN OMO | SODO ZURIA | Gebs | 06-54-00-N | 37-48-00-E | 2330 | Population / Mixture | Spikes | 5 |
| 3898 | Oromiya | ARSSI | GEDEB | Gebs | 07-22-00-N | 39-17-00-E | 3020 | Population / Mixture | Spikes | 6 |
| 3854 | SNNP | SEMEN OMO | BOREDA ABAYA | Gebs | 06-31-00-N | 37-45-00-E | 2350 | Population / Mixture | Spikes | 6 |
| 3851 | SNNP | SEMEN OMO | DAMOT GALE | Gebs | 06-43-00-N | 37-44-00-E | 1790 | Population / Mixture | Spikes | 6 |
| 3904 | Oromiya | ARSSI | SHERKA | Gebs | 07-32-00-N | 39-16-00-E | 2870 | Population / Mixture | Spikes | 5 |
| 3963 | Oromiya | MIRAB HARERGE | TULO | Garbu | 09-16-00-N | 41-08-00-E | 2570 | Population / Mixture | Spikes | 7 |
| 3906 | Oromiya | ARSSI | SHERKA | Gebs | 07-32-00-N | 39-16-00-E | 2860 | Population / Mixture | Spikes | 5 |
| 3903 | Oromiya | ARSSI | BEKOJI | Gerbu Adi | 07-29-00-N | 39-16-00-E | 2900 | Population / Mixture | Spikes | 6 |
| 3905 | Oromiya | ARSSI | SHERKA | Gebs | 07-32-00-N | 39-16-00-E | 2870 | Population / Mixture | Spikes | 5 |
| 3946 | Oromiya | MIRAB HARERGE | CHIRO | Gebs | 09-03-00-N | 40-55-00-E | 2320 | Population / Mixture | Spikes | 6 |
| 3409 | Oromiya | SEMEN SHEWA | MULONA SULULTA | Magge | 09-33-00-N | 38-51-00-E | 2730 | Single Line | Pods |  |
| 3402 | Oromiya | SEMEN SHEWA | GERAR JARSO | Gayye | 09-33-00-N | 38-52-00-E | 2710 | Single Line | Pods |  |
| 3902 | Oromiya | ARSSI | BEKOJI | Gebs | 07-28-00-N | 39-16-00-E | 3020 | Population / Mixture | Spikes | 7 |
| 3901 | Oromiya | ARSSI | BEKOJI | Gebs Kasale | 07-24-00-N | 39-16-00-E | 3000 | Population / Mixture | Spikes | 6 |
| 3900 | Oromiya | ARSSI | BEKOJI | Gebs | 07-22-00-N | 39-17-00-E | 3020 | Population / Mixture | Spikes | 6 |
| 3853 | SNNP | SEMEN OMO | BOREDA ABAYA | Gebs | 06-33-00-N | 37-41-00-E | 2220 | Population / Mixture | Spikes | 5 |
| 3941 | Oromiya | MIRAB HARERGE | HABRO | Gebs | 08-54-00-N | 40-46-00-E | 1890 | Population / Mixture | Spikes | 6 |
| 3940 | Oromiya | MIRAB HARERGE | CHIRO | Gebs | 08-54-00-N | 40-46-00-E | 1830 | Population / Mixture | Spikes | 6 |
| 3938 | Oromiya | MIRAB HARERGE | CHIRO | Gebs | 09-03-00-N | 40-53-00-E | 2230 | Population / Mixture | Spikes | 7 |
| 3937 | Oromiya | ARSSI | DIGELUNA TIJO | Gebs | 07-40-00-N | 39-00-00-E | 2540 | Population / Mixture | Spikes | 6 |
| 3936 | Oromiya | ARSSI | DIGELUNA TIJO | Key Gebs | 07-38-00-N | 39-14-00-E | 2530 | Population / Mixture | Spikes | 6 |
| 3935 | Oromiya | ARSSI | BEKOJI | Gebs | 07-33-00-N | 39-16-00-E | 2760 | Population / Mixture | Spikes | 6 |
| 3352 | Oromiya | ARSSI | SUDE | Aruso | 08-08-00-N | 39-45-00-E | 2660 |  | Spikes | 5 |
| 3400 | Amara | SEMEN SHEWA | MORETNA JIRU | Damoi | 09-40-00-N | 39-11-00-E | 2670 |  | Pods |  |
| 3397 | Oromiya | SEMEN SHEWA | KEMBIBIT | Falibae | 09-34-00-N | 39-12-00-E | 2710 | Single Line | Pods |  |
| 3396 | Oromiya | SEMEN SHEWA | KEMBIBIT | Falibae | 09-34-00-N | 39-12-00-E | 2710 | Single Line | Pods |  |
| 3395 | Oromiya | SEMEN SHEWA | KEMBIBIT | Mugga | 09-33-00-N | 39-13-00-E | 2710 | Single Line | Pods |  |
| 3394 | Oromiya | SEMEN SHEWA | KEMBIBIT | Magge | 09-31-00-N | 39-13-00-E | 2750 | Single Line | Pods |  |
| 3393 | Oromiya | SEMEN SHEWA | KEMBIBIT | Gerbu Adi | 09-31-00-N | 39-14-00-E | 2790 | Population / Mixture | Spikes |  |
| 3392 | Oromiya | SEMEN SHEWA | KEMBIBIT | Magge | 09-24-00-N | 39-16-00-E | 2880 | Single Line | Pods |  |
| 3391 | Oromiya | SEMEN SHEWA | KEMBIBIT | Gerbu | 09-19-00-N | 39-16-00-E | 2920 | Population / Mixture | Spikes | 2 |
| 3390 | Oromiya | ARSSI | BEKOJI | Gerbu Adi | 07-30-00-N | 39-12-00-E | 2605 | Population / Mixture | Spikes |  |
| 3389 | Oromiya | ARSSI | DIGELUNA TIJO | Gerbu | 07-43-00-N | 39-21-00-E | 3100 | Population / Mixture | Spikes |  |
| 3347 | Oromiya | ARSSI | CHOLE | Mauge aba Mota | 08-12-00-N | 39-54-00-E | 3050 |  | Spikes |  |
| 3346 | Oromiya | ARSSI | CHOLE | Nazo | 08-10-00-N | 39-54-00-E | 2925 |  | Spikes |  |
| 3345 | Oromiya | ARSSI | CHOLE | Kesele | 08-08-00-N | 39-54-00-E | 2760 | Population / Mixture | Spikes |  |
| 3338 | Oromiya | ARSSI | CHOLE | Kesele Temej | 08-13-00-N | 39-55-00-E | 3050 |  | Spikes |  |
| 3337 | Oromiya | ARSSI | CHOLE | Nazo | 08-13-00-N | 39-53-00-E | 2915 |  | Spikes |  |
| 3849 | SNNP | SEMEN OMO | DAMOT GALE | Banga | 06-58-00-N | 37-50-00-E | 2070 | Population / Mixture | Spikes | 6 |
| 3386 | Oromiya | ARSSI | DIGELUNA TIJO | Seman | 07-48-00-N | 39-19-00-E | 2930 | Population / Mixture | Spikes |  |
| 3388 | Oromiya | ARSSI | DIGELUNA TIJO | Gerbu | 07-43-00-N | 39-21-00-E | 3150 | Population / Mixture | Spikes | 5 |
| 3327 | Oromiya | MISRAK SHEWA | ADAMA | Gerbu | 08-34-00-N | 39-16-00-E | 1595 |  |  | 2 |
| 3328 | Oromiya | MISRAK SHEWA | BOSET | Gerbu | 08-39-00-N | 39-38-00-E | 1550 |  | Seed |  |
| 3329 | Oromiya | ARSSI | MERTI |  | 08-23-00-N | 39-44-00-E | 2210 |  | Spikes | 2 |
| 3330 | Oromiya | ARSSI | GOLOLCHA | Nazo Feres Game | 08-22-00-N | 39-53-00-E | 2520 | Population / Mixture | Seed |  |
| 3332 | Oromiya | ARSSI | CHOLE | Nazo | 08-21-00-N | 39-56-00-E | 2840 |  | Spikes |  |
| 3333 | Oromiya | ARSSI | CHOLE | Kinchicho | 08-21-00-N | 39-56-00-E | 3000 |  | Spikes |  |
| 3336 | Oromiya | ARSSI | CHOLE | Aba Moto | 08-13-00-N | 39-53-00-E | 2915 |  | Spikes |  |
| 3335 | Oromiya | ARSSI | CHOLE | Tikur Gebs | 08-14-00-N | 39-53-00-E | 2915 | Population / Mixture | Spikes |  |
| 3942 | Oromiya | MIRAB HARERGE | HABRO | Netella Gebs | 09-05-00-N | 40-50-00-E | 1900 | Population / Mixture | Spikes |  |
| 3410 | Oromiya | SEMEN SHEWA | MULONA SULULTA | Kayl Gebs | 09-19-00-N | 38-44-00-E | 2660 | Single Line | Pods |  |
| 3411 | Oromiya | SEMEN SHEWA | MULONA SULULTA | Nech Gebs | 09-19-00-N | 38-44-00-E | 2660 | Single Line | Pods |  |
| 3412 | Oromiya | SEMEN SHEWA | MULONA SULULTA | Magge | 09-19-00-N | 38-44-00-E | 2660 | Single Line | Pods |  |
| 3413 | Oromiya | SEMEN SHEWA | MULONA SULULTA | Gebes | 09-23-00-N | 38-40-00-E | 2500 | Single Line | Spikes |  |
| 3299 | Oromiya | ARSSI | KOFELE | Aruso | 07-05-00-N | 38-45-00-E | 2720 |  | Spikes | 3 |
| 3298 | Oromiya | ARSSI | KOFELE | Falibaye | 07-04-00-N | 38-49-00-E | 2800 |  | Spikes | 3 |
| 3297 | Oromiya | ARSSI | KOFELE | Falibaye | 07-00-00-N | 39-06-00-E | 1550 |  | Spikes |  |
| 3296 | Oromiya | BALE | ADABA | Hunetaa | 07-03-00-N | 39-24-00-E | 2450 |  | Seed |  |
| 3294 | Oromiya | BALE | ADABA | Magie Guracha | 06-55-00-N | 39-29-00-E | 2830 |  | Spikes |  |
| 3293 | Oromiya | BALE | ADABA | Bahre Seded | 06-55-00-N | 39-29-00-E | 2830 |  | Spikes |  |
| 3357 | Oromiya | ARSSI | SUDE | Aruso | 08-06-00-N | 39-39-00-E | 2680 |  | Spikes |  |
| 3353 | Oromiya | ARSSI | SUDE | Kesele | 07-59-00-N | 39-55-00-E | 2580 |  | Spikes |  |
| 3359 | Oromiya | ARSSI | SUDE |  | 08-05-00-N | 39-38-00-E | 2660 |  | Spikes | 3 |
| 3361 | Oromiya | ARSSI | SUDE | Aruso | 08-12-00-N | 39-34-00-E | 2650 |  | Spikes | 2 |
| 3360 | Oromiya | ARSSI | SUDE | Kesele | 08-03-00-N | 39-33-00-E | 2700 |  | Spikes |  |
| 3292 | Oromiya | BALE | ADABA | Kasalee | 06-58-00-N | 39-24-00-E | 2630 |  | Seed |  |
| 3968 | Oromiya | MISRAK HARERGE | DEDER | Garbu | 09-17-00-N | 41-29-00-E | 2150 | Population / Mixture | Spikes | 7 |
| 3907 | Oromiya | ARSSI | SHERKA | Gebs | 07-32-00-N | 39-16-00-E | 2860 | Population / Mixture | Spikes | 5 |
| 3446 | Oromiya | MIRAB SHEWA | DENDI | Garbu Adi | 09-13-00-N | 38-09-00-E | 2960 | Single Line | Pods |  |
| 3445 | Oromiya | MIRAB SHEWA | DENDI | Shamare | 09-11-00-N | 38-11-00-E | 3020 | Single Line | Pods |  |
| 3443 | Oromiya | MIRAB SHEWA | DENDI | Bakemi | 09-07-00-N | 38-12-00-E | 2990 | Single Line | Pods |  |
| 3444 | Oromiya | MIRAB SHEWA | DENDI | Garbu Adi | 09-11-00-N | 38-11-00-E | 3020 | Single Line | Pods |  |
| 3442 | Oromiya | MIRAB SHEWA | DENDI | Gerbu Guracha | 09-06-00-N | 38-12-00-E | 2900 | Single Line | Pods |  |
| 3441 | Oromiya | MIRAB SHEWA | DENDI | Shamari | 09-06-00-N | 38-12-00-E | 2900 | Single Line | Pods |  |
| 3440 | Oromiya | MIRAB SHEWA | DENDI | Samareta | 09-03-00-N | 38-07-00-E | 2393 | Single Line | Pods | 6 |
| 3941 | Oromiya | MIRAB HARERGE | HABRO | Gebs | 08-54-00-N | 40-46-00-E | 1890 | Population / Mixture | Spikes | 6 |
| 3934 | Oromiya | ARSSI | BEKOJI | Gebs | 07-33-00-N | 39-16-00-E | 2760 | Population / Mixture | Spikes | 6 |
| 3932 | Oromiya | ARSSI | BEKOJI |  | 07-32-00-N | 39-16-00-E | 2860 | Population / Mixture | Spikes | 6 |
| 3835 | SNNP | SIDAMA | SHEBEDINO |  | 06-54-00-N | 37-46-00-E | 2350 | Population / Mixture | Spikes | 5 |
| 3836 | SNNP | SEMEN OMO | SODO ZURIA | Banga | 06-53-00-N | 37-46-00-E | 2235 | Population / Mixture | Spikes | 5 |
| 3913 | Oromiya | ARSSI | SHERKA | Gebs | 07-34-00-N | 39-29-00-E | 2640 | Population / Mixture | Spikes | 6 |
| 3915 | Oromiya | ARSSI | SHERKA | Gebs | 07-35-00-N | 39-25-00-E | 2620 | Population / Mixture | Spikes | 6 |
| 3931 | Oromiya | ARSSI | BEKOJI | Gebs | 07-32-00-N | 39-16-00-E | 2860 | Population / Mixture | Spikes | 6 |
| 3927 | Oromiya | ARSSI | ROBE | Kasale | 07-34-00-N | 39-29-00-E | 2660 | Population / Mixture | Spikes | 5 |
| 3956 | Oromiya | MIRAB HARERGE | TULO | Fato | 08-57-00-N | 40-50-00-E | 2300 | Population / Mixture | Spikes | 7 |
| 3834 | SNNP | SIDAMA | SHEBEDINO | Banta | 06-53-00-N | 37-46-00-E | 2210 | Population / Mixture | Spikes | 6 |
| 3933 | Oromiya | ARSSI | BEKOJI | Gebs | 07-33-00-N | 39-16-00-E | 2780 | Population / Mixture | Spikes | 5 |
| 3926 | Oromiya | ARSSI | SHERKA | Netch Gebs | 07-33-00-N | 39-29-00-E | 2730 | Population / Mixture | Spikes | 6 |
| 3958 | Oromiya | MIRAB HARERGE | TULO | Fato | 08-57-00-N | 40-50-00-E | 2280 | Population / Mixture | Spikes | 7 |
| 3824 | Amara | DEBUB GONDAR | FARTA | Bule Gebs | 11-49-00-N | 38-14-00-E | 2900 |  | Spikes |  |
| 3959 | Oromiya | MIRAB HARERGE | TULO | Gardu Adi | 09-15-00-N | 41-07-00-E | 2420 | Population / Mixture | Spikes | 7 |
| 3960 | Oromiya | MIRAB HARERGE | TULO | Garbu | 09-15-00-N | 41-08-00-E | 2380 | Population / Mixture | Spikes | 7 |
| 3855 | SNNP | SEMEN OMO | CHENCHA | Gebs | 06-28-00-N | 37-39-00-E | 2430 | Population / Mixture | Spikes | 6 |
| 3916 | Oromiya | ARSSI | SHERKA | Gebs | 07-36-00-N | 39-28-00-E | 2600 | Population / Mixture | Spikes | 6 |
| 3899 |  |  |  | Gebs | 07-22-00-N | 39-17-00-E | 3020 | Population / Mixture | Spikes | 6 |
| 3887 | Oromiya | ARSSI | KOFELE | Aruso | 07-06-00-N | 38-50-00-E | 2630 | Population / Mixture | Spikes | 6 |
| 3886 | Oromiya | ARSSI | KOFELE | Aruso | 07-06-00-N | 38-48-00-E | 2650 | Population / Mixture | Spikes | 6 |
| 3976 | Amara | DEBUB WELLO | DESSIE ZURIA | Gebs | 11-08-00-N | 39-53-00-E | 1960 | Population / Mixture | Spikes | 7 |
| 3888 | Oromiya | ARSSI | KOFELE | Aruso | 07-06-00-N | 38-50-00-E | 2630 | Population / Mixture | Spikes | 6 |
| 3505 | Amara | MIRAB GOJAM | DEMBECHA | Mesno Gebs | 10-37-00-N | 34-25-00-E | 1880 | Single Line | Spikes | 6 |
| 3565 | Benishangul Gumuz | METEKEL | WENBERA | Kemedi | 10-30-00-N | 35-50-00-E | 2700 | Single Line | Spikes |  |
| 3925 | Oromiya | ARSSI | SHERKA | Gebs Kesele | 07-33-00-N | 39-29-00-E | 2730 | Population / Mixture | Spikes | 6 |
| 3962 | Oromiya | MIRAB HARERGE | TULO | Garbu | 09-16-00-N | 41-08-00-E | 2570 | Population / Mixture | Spikes | 7 |
| 3924 | Oromiya | ARSSI | SHERKA | Gebs Aruso | 07-33-00-N | 39-29-00-E | 2730 | Population / Mixture | Spikes | 6 |
| 3504 | Amara | AGEW AWI | BANJA | Gebs | 11-02-00-N | 36-54-00-E | 2540 | Single Line | Spikes | 3 |
| 3500 | Amara | DEBUB GONDAR | ESTE | Gebes | 11-38-00-N | 38-04-00-E | 2620 |  | Spikes | 6 |
| 3499 | Amara | DEBUB GONDAR | FARTA | Kinkena | 11-49-00-N | 38-13-00-E | 2810 | Single Line | Spikes | 6 |
| 3486 | Amara | AGEW AWI | BANJA | Gebs | 10-50-00-N | 37-12-00-E | 2560 | Single Line | Spikes | 5 |
| 3484 | Amara | MIRAB GOJAM | SEKELA | Salimi | 10-58-00-N | 37-13-00-E | 2780 | Single Line | Spikes | 5 |
| 3483 | Amara | MIRAB GOJAM | SEKELA | Gebsa | 10-58-00-N | 37-13-00-E | 2880 |  |  | 5 |
| 3482 | Amara | AGEW AWI | BANJA | Wontaka | 10-55-00-N | 37-13-00-E | 2900 | Population / Mixture | Spikes | 7 |
| 3481 | Amara | AGEW AWI | BANJA | Salimi | 10-53-00-N | 37-14-00-E | 2750 | Single Line | Spikes |  |
| 3487 | Amara | AGEW AWI | BANJA | Sinde Meno | 10-59-00-N | 36-56-00-E | 2555 | Single Line | Spikes | 7 |
| 3348 | Oromiya | ARSSI | CHOLE | Kesele | 08-12-00-N | 39-54-00-E | 3050 |  | Seed |  |
| 3472 | Amara | MIRAB GOJAM | JABI TEHNAN | Gebsa | 10-45-00-N | 37-06-00-E | 2350 | Single Line | Spikes | 7 |
| 3464 | Oromiya | MIRAB SHEWA | DENDI | Samareta | 09-00-00-N | 38-04-00-E | 2420 | Single Line | Seed | 6 |
| 3462 | Oromiya | SEMEN SHEWA | BEREHNA ALELTU | Gebs | 09-02-00-N | 38-55-00-E | 2520 | Single Line | Seed |  |
| 3451 | Oromiya | SEMEN SHEWA | MULONA SULULTA | Balami | 09-05-00-N | 38-47-00-E | 3020 | Single Line | Pods |  |
| 3291 | Oromiya | BALE | ADABA | Bahre Seded | 06-58-00-N | 06-58-00-E | 2630 |  | Spikes |  |
| 3290 | Oromiya | BALE | ADABA | Garbu Dima | 06-59-00-N | 39-23-00-E | 2470 | Population / Mixture | Spikes |  |
| 3287 | Oromiya | BALE | GOBA | Bahre Seded | 07-02-00-N | 39-56-00-E | 2750 | Population / Mixture | Spikes | 3 |
| 3351 | Oromiya | ARSSI | CHOLE | Gerbu | 08-15-00-N | 39-47-00-E | 2810 |  | Spikes | 5 |
| 3350 | Oromiya | ARSSI | CHOLE | Kinchicho | 08-13-00-N | 39-55-00-E | 3080 |  |  |  |
| 3289 | Oromiya | BALE | GOBA |  | 06-57-00-N | 39-59-00-E | 2840 | Population / Mixture | Spikes |  |
| 3933 | Oromiya | ARSSI | BEKOJI | Gebs | 07-33-00-N | 39-16-00-E | 2780 | Population / Mixture | Spikes | 5 |
| 3288 | Oromiya | BALE | GOBA | Kinchicho | 07-00-00-N | 39-53-00-E | 2820 | Population / Mixture | Spikes |  |
| 3363 | Oromiya | ARSSI | SUDE | Aruso | 07-59-00-N | 39-41-00-E | 2580 |  | Spikes | 2 |
| 3366 | Oromiya | ARSSI | SUDE | Gerbu Adi | 07-53-00-N | 39-44-00-E | 2520 |  | Spikes |  |
| 3365 | Oromiya | ARSSI | SUDE | Aruso | 07-58-00-N | 39-41-00-E | 2500 | Population / Mixture | Spikes |  |
| 3362 | Oromiya | ARSSI | SUDE | Aruso | 08-19-00-N | 39-43-00-E | 2690 |  | Spikes |  |
| 3349 | Oromiya | ARSSI | CHOLE | Nazo | 08-12-00-N | 39-54-00-E | 3050 | Population / Mixture | Spikes |  |
| 3300 | Oromiya | ARSSI | KOFELE | Samarieta | 07-05-00-N | 38-45-00-E | 2720 |  | Spikes |  |
| 3866 | SNNP | BENCH MAJI | DIRASHE SPECIAL WERE | Gebs | 05-39-00-N | 37-23-00-E | 2020 | Population / Mixture | Spikes | 6 |
| 3450 | Oromiya | MIRAB SHEWA | JELDU | Garbu Addicho | 09-18-00-N | 38-04-00-E | 2810 | Single Line | Pods |  |
| 3449 | Oromiya | MIRAB SHEWA | JELDU | Balami Adi | 09-18-00-N | 38-04-00-E | 2850 | Single Line | Pods |  |
| 3448 | Oromiya | MIRAB SHEWA | JELDU | Mugga | 09-18-00-N | 38-04-00-E | 2850 | Single Line | Pods |  |
| 3447 | Oromiya | MIRAB SHEWA | DENDI | Samareta | 09-13-00-N | 38-09-00-E | 2960 | Single Line | Pods |  |
| 3382 | Oromiya | ARSSI | DIGELUNA TIJO | Gerbu Guracha | 07-45-00-N | 39-15-00-E | 2630 | Population / Mixture | Spikes |  |
| 3880 | Oromiya | ARSSI | KOFELE | Gebs | 07-09-00-N | 38-52-00-E | 2760 | Population / Mixture | Spikes | 5 |
| 3965 | Oromiya | MISRAK HARERGE | GORO GUTU | Garbu | 09-23-00-N | 41-23-00-E | 2320 | Population / Mixture | Spikes | 7 |
| 3879 | Oromiya | ARSSI | KOFELE | Gebs | 07-09-00-N | 38-52-00-E | 2760 | Population / Mixture | Spikes | 5 |
| 2878 |  |  |  |  |  |  |  |  |  |  |
| 3877 | Oromiya | ARSSI | KOFELE | Gebs | 07-09-00-N | 38-28-00-E | 2790 | Population / Mixture | Spikes | 6 |
| 3964 | Oromiya | MIRAB HARERGE | TULO | Garbu | 09-17-00-N | 41-13-00-E | 2500 | Population / Mixture | Spikes | 7 |
| 3876 | Oromiya | ARSSI | KOFELE | Arsi | 07-08-00-N | 38-49-00-E | 2750 | Population / Mixture | Spikes | 6 |
| 3875 | Oromiya | ARSSI | KOFELE | Gebs | 07-06-00-N | 27-48-00-E | 2730 | Population / Mixture | Spikes | 6 |
| 3874 | Oromiya | ARSSI | KOFELE | Garbu | 07-06-00-N | 27-48-00-E | 2730 | Population / Mixture | Spikes |  |
| 3867 | SNNP | BENCH MAJI | DIRASHE SPECIAL WERE | Gebs | 05-39-00-N | 37-23-00-E | 2020 | Population / Mixture | Spikes | 6 |
| 3873 | Oromiya | MISRAK SHEWA | SHASHEMENE | Garbu | 07-11-00-N | 38-37-00-E | 1980 | Population / Mixture | Spikes | 6 |
| 3864 | SNNP | SEMEN OMO | CHENCHA | Gebs | 06-10-00-N | 37-33-00-E | 2700 | Population / Mixture | Spikes | 6 |
| 3863 | SNNP | SEMEN OMO | CHENCHA | Gebs | 06-10-00-N | 37-33-00-E | 2700 | Population / Mixture | Spikes | 6 |
| 3862 | SNNP | SEMEN OMO | CHENCHA | Gebs | 06-10-00-N | 37-33-00-E | 2700 | Population / Mixture | Spikes | 6 |
| 3321 | Oromiya | ARSSI | CHOLE | Nazo | 08-21-00-N | 39-56-00-E | 3000 |  | Spikes |  |
| 3380 | Oromiya | ARSSI | DIGELUNA TIJO | Gebu Guracha | 07-42-00-N | 39-11-00-E | 2535 | Population / Mixture | Spikes |  |
| 3476 | Amara | AGEW AWI | BANJA | Salmie | 10-52-00-N | 37-07-00-E | 2580 | Single Line | Spikes | 5 |
| 3285 | Oromiya | BALE | GINIR | Kesela | 07-22-00-N | 40-14-00-E | 2480 | Population / Mixture | Spikes | 5 |
| 3284 | Oromiya | BALE | GINIR | Mooga | 07-22-00-N | 40-14-00-E | 2480 | Population / Mixture | Spikes | 5 |
| 3283 | Oromiya | BALE | GASERANA GOLOLCHA | Sindie Mana Kin | 07-25-00-N | 40-24-00-E | 2410 | Population / Mixture | Spikes | 3 |
| 3282 | Oromiya | BALE | GINIR | Kinchicho | 07-25-00-N | 40-23-00-E | 2360 | Population / Mixture | Spikes | 3 |
| 3368 | Oromiya | ARSSI | SUDE | Aruso | 07-48-00-N | 39-50-00-E | 2500 | Population / Mixture | Spikes | 2 |
| 3368 | Oromiya | ARSSI | SUDE | Aruso | 07-48-00-N | 39-50-00-E | 2500 | Population / Mixture | Spikes | 2 |
| 3910 | Oromiya | ARSSI | SHERKA | Gebs | 07-35-00-N | 39-16-00-E | 2750 | Population / Mixture | Spikes | 6 |
| 3882 | Oromiya | ARSSI | KOFELE | Gebs | 07-11-00-N | 38-53-00-E | 2790 | Population / Mixture | Spikes | 5 |
| 3908 | Oromiya | ARSSI | SHERKA | Gebs | 07-35-00-N | 39-26-00-E | 2855 | Population / Mixture | Spikes | 6 |
| 3378 | Oromiya | ARSSI | ROBE | workie | 07-31-00-N | 40-03-00-E | 2450 | Population / Mixture | Spikes | 2 |
| 3923 | Oromiya | ARSSI | SHERKA | Gebs Mugga | 07-35-00-N | 39-28-00-E | 2820 | Population / Mixture | Spikes | 6 |
| 3278 | Oromiya | BALE | GASERANA GOLOLCHA | Kinchicho | 07-40-00-N | 40-38-00-E | 2090 | Population / Mixture | Spikes | 3 |
| 3320 | Oromiya | ARSSI | CHOLE | Workie | 08-21-00-N | 39-56-00-E | 2840 |  | Spikes |  |
| 3319 | Oromiya | ARSSI | CHOLE | Mauge | 08-22-00-N | 39-56-00-E | 2720 |  | Spikes | 2 |
| 3917 | Oromiya | ARSSI | SHERKA | Gebs | 07-38-00-N | 39-30-00-E | 2380 | Population / Mixture | Spikes | 6 |
| 3318 | Oromiya | ARSSI | CHOLE | Nazo | 08-22-00-N | 39-56-00-E | 2720 | Population / Mixture | Spikes | 2 |
| 3510 | Amara | MIRAB GOJAM | DEGA DAMOT | Seter | 10-51-00-N | 37-34-00-E | 2770 | Population / Mixture | Spikes | 7 |
| 3509 | Amara | MIRAB GOJAM | DEGA DAMOT | Jibe Sed | 10-51-00-N | 37-34-00-E | 2770 | Population / Mixture | Spikes | 5 |
| 3317 | Oromiya | ARSSI | CHOLE |  | 08-22-00-N | 39-56-00-E | 2720 | Population / Mixture | Spikes | 2 |
| 3308 | Oromiya | ARSSI | KOFELE |  | 07-00-00-N | 38-36-00-E | 2430 |  | Spikes |  |
| 3975 | Amara | OROMIYA | BATI | Gebs | 11-08-00-N | 39-53-00-E | 1960 | Population / Mixture | Seed | 7 |
| 3977 | Amara | DEBUB WELLO | DESSIE ZURIA | Gebs | 11-12-00-N | 39-40-00-E | 2530 | Population / Mixture | Spikes | 7 |
| 3922 | Oromiya | ARSSI | SHERKA | Aruso | 07-36-00-N | 39-28-00-E | 2590 | Population / Mixture | Spikes | 5 |
| 3961 | Oromiya | MIRAB HARERGE | TULO | Gardu Kakalo | 09-15-00-N | 41-04-00-E | 2450 | Population / Mixture | Spikes | 7 |
| 3861 | SNNP | SEMEN OMO | CHENCHA | Gebs | 06-10-00-N | 37-33-00-E | 2700 | Population / Mixture | Spikes | 6 |
| 3860 | SNNP | SEMEN OMO | CHENCHA | Gebs | 06-10-00-N | 37-33-00-E | 2680 | Population / Mixture | Spikes | 7 |
| 3279 | Oromiya | BALE | GASERANA GOLOLCHA | Garboo | 07-32-00-N | 40-40-00-E | 1985 |  | Spikes | 3 |
| 3856 | SNNP | SEMEN OMO | CHENCHA | Banga | 06-16-00-N | 37-34-00-E | 2820 | Population / Mixture | Spikes | 5 |
| 3857 | SNNP | SEMEN OMO | CHENCHA | Gebs | 06-14-00-N | 37-35-00-E | 2850 | Population / Mixture | Spikes | 6 |
| 3859 | SNNP | SEMEN OMO | CHENCHA | Gebs | 06-12-00-N | 37-34-00-E | 2970 | Population / Mixture | Spikes | 6 |
| 3858 | SNNP | SEMEN OMO | CHENCHA | Gebs | 06-11-00-N | 37-36-00-E | 2900 | Population / Mixture | Spikes | 6 |
| 3314 | Oromiya | ARSSI | CHOLE | Kinchicho | 08-23-00-N | 39-56-00-E | 2710 |  | Spikes |  |
| 3313 | Oromiya | ARSSI | MERTI |  | 08-23-00-N | 39-44-00-E | 2210 |  | Spikes |  |
| 3277 | Oromiya | BALE | GASERANA GOLOLCHA | Letena | 07-40-00-N | 40-42-00-E | 2220 |  | Spikes | 3 |
| 3316 | Oromiya | ARSSI | CHOLE |  | 08-22-00-N | 39-56-00-E | 2720 |  | Spikes | 2 |
| 3315 | Oromiya | ARSSI | CHOLE | Deboye | 08-23-00-N | 39-56-00-E | 2630 |  | Spikes |  |
| 3507 | Amara | MIRAB GOJAM | DEMBECHA | Awra Gebs | 10-37-00-N | 37-29-00-E | 2415 | Single Line | Spikes | 6 |
| 3506 | Amara | MIRAB GOJAM | DEMBECHA | Mesno Gebs | 10-31-00-N | 37-27-00-E | 2100 | Single Line | Spikes | 6 |
| 3312 | Oromiya | ARSSI | MERTI | Gerbu | 08-23-00-N | 39-44-00-E | 2275 |  | Seed |  |
| 3381 | Oromiya | ARSSI | DIGELUNA TIJO | Worki | 07-45-00-N | 39-15-00-E | 2630 | Population / Mixture | Spikes |  |
| 3881 | Oromiya | ARSSI | KOFELE | Gebs | 07-11-00-N | 38-53-00-E | 2790 | Population / Mixture | Spikes | 5 |
| 3384 | Oromiya | ARSSI | DIGELUNA TIJO | Gerbu Adi | 07-45-00-N | 39-16-00-E | 2750 | Population / Mixture | Spikes |  |
| 3883 | Oromiya | ARSSI | KOFELE | Gebs | 07-14-00-N | 38-57-00-E | 2850 | Population / Mixture | Spikes | 6 |
| 3419 | Oromiya | SEMEN SHEWA | MULONA SULULTA | Balemi | 09-05-00-N | 38-37-00-E | 2680 | Population / Mixture | Pods |  |
| 3379 | Oromiya | ARSSI | DIGELUNA TIJO | Gebu Adi | 07-42-00-N | 39-11-00-E | 2535 | Population / Mixture | Spikes |  |
| 3885 | Oromiya | ARSSI | KOFELE | Ausso | 07-04-00-N | 38-48-00-E | 2680 | Population / Mixture | Spikes | 6 |
| 3884 | Oromiya | ARSSI | KOFELE | Gebs | 07-14-00-N | 38-57-00-E | 2850 | Population / Mixture | Spikes | 6 |
| 3322 | Oromiya | ARSSI | CHOLE | Nazo | 08-21-00-N | 39-56-00-E | 3000 |  | Spikes | 1 |
| 3323 | Oromiya | ARSSI | CHOLE | Aba Moto | 08-18-00-N | 39-55-00-E | 2870 |  | Seed |  |
| 3911 | Oromiya | ARSSI | SHERKA | Gebs | 07-35-00-N | 39-25-00-E | 2640 | Population / Mixture | Spikes | 6 |
| 3324 | Oromiya | ARSSI | CHOLE | Feres Game | 08-16-00-N | 39-55-00-E | 2830 |  | Spikes |  |
| 3325 | Oromiya | ARSSI | CHOLE | Aba Moto Mauge | 08-13-00-N | 39-55-00-E | 3050 | Population / Mixture | Spikes |  |
| 3326 | Oromiya | ARSSI | CHOLE | Kinchicho | 08-13-00-N | 39-55-00-E | 3050 |  | Spikes |  |
| 3310 | Oromiya | ARSSI | KOFELE | Shasho-Aruso | 07-11-00-N | 38-30-00-E | 2480 |  | Spikes | 3 |
| 3842 | SNNP | SEMEN OMO | SODO ZURIA | Banga | 06-53-00-N | 37-50-00-E | 2000 | Population / Mixture | Spikes | 6 |
| 3955 | Oromiya | MIRAB HARERGE | TULO | Fato | 08-59-00-N | 40-51-00-E | 2310 | Population / Mixture | Spikes | 7 |
| 3848 | SNNP | SEMEN OMO | BOLOSO SORE | Gebs | 06-59-00-N | 37-53-00-E | 2050 | Population / Mixture | Spikes | 6 |
| 3367 | Oromiya | ARSSI | SUDE | Nech Gebs | 07-54-00-N | 39-47-00-E | 2500 |  | Spikes | 2 |
| 3375 | Oromiya | ARSSI | ROBE | Arusso | 07-50-00-N | 39-44-00-E | 2470 |  | Spikes | 2 |
| 3374 | Oromiya | ARSSI | SUDE | Arusso | 07-41-00-N | 39-58-00-E | 2500 |  | Spikes |  |
| 3949 | Oromiya | MIRAB HARERGE | CHIRO | Gebs | 09-01-00-N | 40-54-00-E | 2290 | Population / Mixture | Spikes | 6 |
| 3947 | Oromiya | MIRAB HARERGE | CHIRO | Gebs | 09-03-00-N | 40-55-00-E | 2310 | Population / Mixture | Spikes | 6 |
| 3909 | Oromiya | ARSSI | SHERKA | Gebs | 07-35-00-N | 39-16-00-E | 2750 | Population / Mixture | Spikes | 6 |
| 3972 | Oromiya | MISRAK HARERGE | META | Garbu | 09-24-00-N | 41-35-00-E | 2200 | Population / Mixture | Spikes |  |
| 3843 | SNNP | SEMEN OMO | SODO ZURIA | Banga | 06-55-00-N | 37-48-00-E | 2270 | Population / Mixture | Spikes | 5 |
| 3373 | Oromiya | ARSSI | SERU | Kinchicho | 07-40-00-N | 40-11-00-E | 2500 |  | Spikes |  |
| 3371 | Oromiya | ARSSI | SUDE | Arusso | 07-49-00-N | 39-55-00-E | 2500 |  | Spikes | 2 |
| 3845 | SNNP | SEMEN OMO | DAMOT GALE | Banga | 06-54-00-N | 37-50-00-E | 2150 | Population / Mixture | Spikes | 6 |
| 3847 | SNNP | SEMEN OMO | DAMOT GALE | Gebs | 06-59-00-N | 37-53-00-E | 2050 | Population / Mixture | Spikes | 6 |
| 3370 | Oromiya | ARSSI | SUDE | Gerbu Bona | 07-48-00-N | 39-55-00-E | 2455 | Population / Mixture | Spikes |  |
| 3369 | Oromiya | ARSSI | SUDE | Gerbu Guracha | 07-48-00-N | 39-54-00-E | 2520 |  | Spikes | 2 |
| 3971 | Oromiya | MISRAK HARERGE | META | Garbu | 09-23-00-N | 41-34-00-E | 2380 | Population / Mixture | Spikes |  |
| 3970 | Oromiya | MISRAK HARERGE | META | Garbu | 09-24-00-N | 41-33-00-E | 2430 | Population / Mixture | Spikes | 7 |
| 3376 | Oromiya | ARSSI | ROBE | Feyi-Bai | 07-45-00-N | 39-40-00-E | 2400 | Population / Mixture | Spikes | 3 |
| 3305 | Oromiya | ARSSI | KOFELE | Mallo | 07-08-00-N | 38-42-00-E | 2570 |  | Spikes | 3 |
| 3377 | Oromiya | ARSSI | ROBE | Arusso | 07-32-00-N | 39-59-00-E | 2445 | Population / Mixture | Spikes | 2 |
| 3969 | Oromiya | MISRAK HARERGE | DEDER | Garbu | 09-23-00-N | 41-31-00-E | 2230 | Population / Mixture | Spikes | 7 |
| 3967 | Oromiya | MISRAK HARERGE | DEDER | Garbu | 09-22-00-N | 41-28-00-E | 2200 | Population / Mixture | Spikes |  |
| 3304 | Oromiya | ARSSI | KOFELE | Wargee | 07-06-00-N | 38-44-00-E | 2630 |  | Spikes |  |
| 3890 | Oromiya | ARSSI | KOFELE | Gebs | 07-01-00-N | 38-53-00-E | 2585 | Population / Mixture | Spikes | 5 |
| 3897 | Oromiya | ARSSI | GEDEB |  | 07-08-00-N | 39-18-00-E | 2500 | Population / Mixture | Spikes | 6 |
| 3420 | Oromiya | SEMEN SHEWA | MULONA SULULTA | Balemi | 09-07-00-N | 38-36-00-E | 2730 | Population / Mixture | Pods |  |
| 3439 | Oromiya | MIRAB SHEWA | DENDI | Samareta | 09-02-00-N | 38-09-00-E | 2372 | Single Line | Pods | 6 |
| 3301 | Oromiya | ARSSI | KOFELE | Aruao guracha | 07-05-00-N | 38-45-00-E | 2710 |  | Spikes | 3 |
| 3302 | Oromiya | ARSSI | KOFELE | 63 | 07-06-00-N | 38-44-00-E | 2630 |  | Spikes | 3 |
| 3889 | Oromiya | ARSSI | KOFELE | Gebs | 07-02-00-N | 38-50-00-E | 2640 | Population / Mixture | Spikes | 5 |
